# Cyclover-Assisted Liquid-Phase Peptide Synthesis Using T3P® as a Green Coupling Reagent

**DOI:** 10.1021/acs.orglett.6c00353

**Published:** 2026-02-24

**Authors:** Priyanka Kushwaha, Marvin Mantel, Peter Talbiersky, Yongfu Li, Anamika Sharma, Beatriz G. de la Torre, Fernando Albericio

**Affiliations:** † Peptide Science Laboratory, School of Chemistry and Physics, 56394 University of KwaZulu-Natal, Westville, Durban 4000, South Africa; ‡ 474231 Curia Germany GmbH, Industriepark Höchst D569, 65926 Frankfurt am Main, Germany; § Biotide Core, LLC, 33815 SE Eastgate Circle, Corvallis, Oregon 97333, United States; ∥ School of Laboratory Medicine and Medical Sciences, College of Health Sciences, University of KwaZulu-Natal, Durban 4041, South Africa; ⊥ Department of Inorganic and Organic Chemistry, University of Barcelona, 08026 Barcelona, Spain

## Abstract

Liquid-phase peptide synthesis (LPPS) continues to evolve toward more sustainable and efficient methodologies. In this study, we introduce Cyclover as a tag that enables efficient, tunable LPPS in 2Me-THF using propylphosphonic anhydride (T3P®) as a coupling reagent. The extraction protocol reduces the PMI ∼2.7-fold while maintaining high purity and isolated yield, offering a greener and versatile route to complex peptides

## Graphical abstract

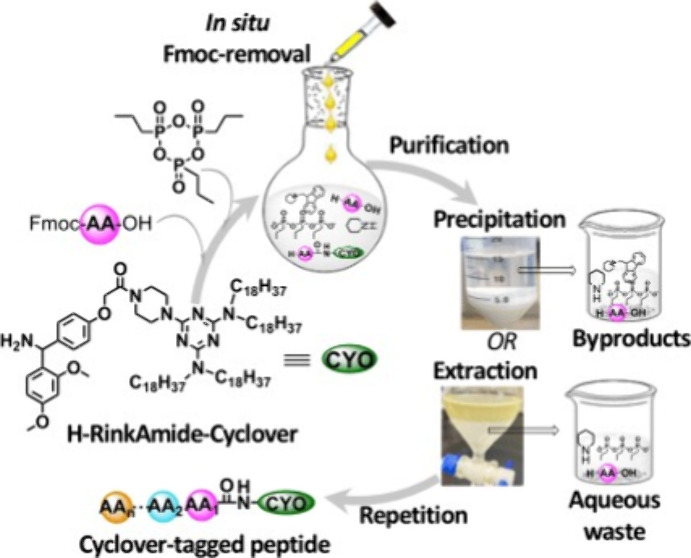

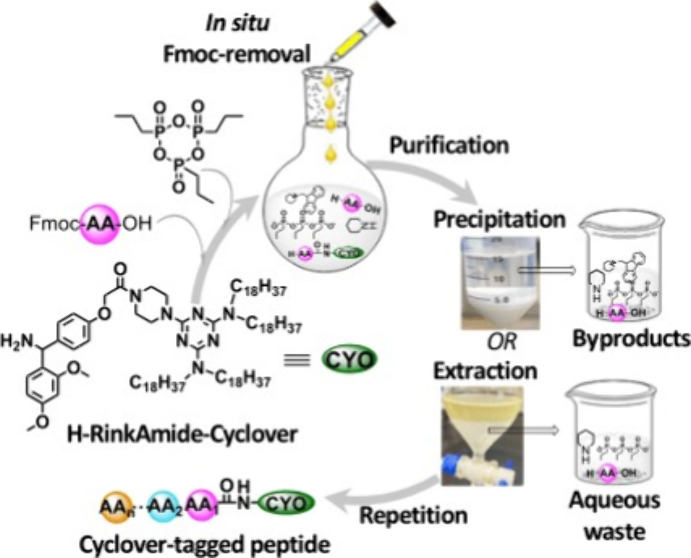

Peptide chemistry has undergone significant development in recent decades, reflecting the growing recognition of peptides as powerful therapeutic agents.^1,2^ Peptide synthesis continues to pose significant challenges in terms of sustainability.^3^ The classical solution peptide synthesis (CSPS) method offers high reaction efficiency but requires labor-intensive chromatographic purification.^4,5^ Solid-phase peptide synthesis (SPPS) simplifies purification yet demands large excesses of reagents and remains the predominant industrial method.^6,7^ Liquid-phase peptide synthesis (LPPS) has re-emerged as a sustainable alternative for large-scale applications,^8^ featuring improved green metrics and reduced waste generation, resulting in a substantial reduction in the process mass intensity (PMI) and complete environmental factor (cEF).^8–11^ This strategy can be considered a mix of CSPS and SPPS, keeping the advantages of both. Thus, the reactions occur in solution, with the growing peptide sequence attached to a tag, which modulates the peptide’s properties. This allows the use of only a slight excess of protected amino acids and operation in a continuous mode, without requiring the isolation and characterization of intermediates, as in CSPS. In contrast to the filtration-based workup in SPPS, LPPS relies on simpler precipitation or extraction manipulation steps. Since the use of the fluorenylmethoxycarbonyl (Fmoc) protecting group is unavoidable due to the lack of commercially available alternatives, the tag and the coupling reagent become the two most critical parameters in LPPS.^3,10,11^ Although many different tags have been reported in the literature,^8^ the most commonly used tags are membrane-enhanced peptide synthesis (MEPS),^12,13^ polycarbon,^14–16^ hydrophobic polymers,^17,18^ and phosphorus-based tags using group-assisted purification (GAP).^19–21^

One drawback associated with the LPPS technology is that most reported tags are proprietary and not commercially available. In this study, we use a new tag, Cyclover [2-(4′-piperazino)-4,6-di­[*N,N*-di­(*n*-octadecyl)­amino]-1,3,5-triazine] (Figure 1), which can be considered a polycarbon tag.^22^ Cyclover is used in combination with T3P® (propylphosphonic anhydride),^23^ a coupling reagent previously reported by Tolomelli and Cabri in light of (liquid-phase) peptide synthesis.^24^ T3P® offers mild reaction conditions with minimal racemization.^25^ An additional advantage of T3P® is the formation of a water-soluble propylphosphonic acid byproduct, which can be readily removed by aqueous extraction, thereby streamlining isolation.^24,26^

**Figure 1:**
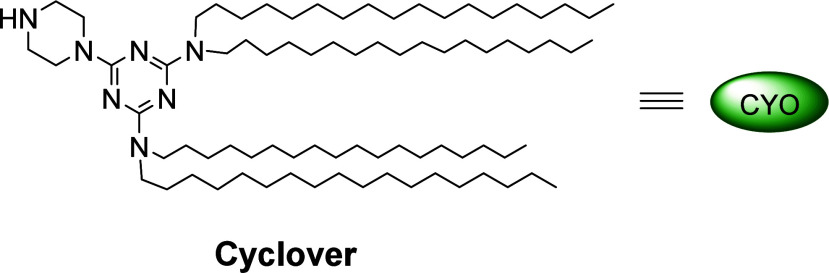
Cyclover tag for LPPS.

In this context, T3P®, which could be considered a trace free coupling reagent, has been proposed by the ACS Green Chemistry Institute Pharmaceutical Roundtable as one of the greenest coupling reagents for amide bond formation.^10^ T3P® furthermore offers greater synthetic convenience than carbodiimides, uronium-, phosphonium-, and guanidinium-based coupling reagents, as those commonly produce water-insoluble products that are not easily removed through standard extraction procedures.^27^ On the other hand, water-soluble 1-ethyl-3-(3-(dimethylamino)­propyl)­carbodiimide (EDC·HCl) showed poor solubility in the organic solvents that are compatible with LPPS. T3P® is commercialized as a 50% (w/w) solution in a wide range of different solvents, including *N,N*-dimethylformamide (DMF), dichloromethane (DCM), 2-methyltetrahydrofuran (2Me-THF), tetrahydrofuran (THF), ethyl acetate (EtOAc), and butyl acetate, at an affordable price and is quite stable.^26^

Cyclover dissolves readily in nonpolar solvents such as cyclohexane, anisole, toluene, 2Me-THF, cyclopentylmethyl ether (CPME), and DCM but precipitates with polar solvents like water, methanol (MeOH), acetonitrile (ACN), EtOAc, or acetone. This tunable solubility enables straightforward product isolation by a simple coupling–precipitation/extraction cycle, thereby eliminating the need for chromatographic purification and providing a scalable and resource-efficient approach to peptide synthesis.

Cyclover was synthesized from cyanuric chloride through a two-step nucleophilic substitution reaction as reported in the literature.^22^ In the current work, the Cyclover tag has been used for peptide synthesis using T3P® as a coupling reagent in the presence of a base employing 2Me-THF as the solvent. The obtained Cyclover was then efficiently coupled with the RinkAmide linker (1.2 equiv) using T3P® (4 equiv) and diisopropylethylamine (DIEA) (8 equiv) in 2Me-THF (2 mL) at room temperature. The coupling reaction proceeded smoothly and was completed within 30 min, as confirmed by TLC (see the Supporting Information). A small portion of the reaction mixture was taken and precipitated with ACN. The precipitate was analyzed by TLC and HPLC to confirm the complete consumption of Cyclover, yielding Fmoc-RinkAmide-Cyclover (see the Supporting Information). Fmoc removal can be performed as a separate step, which implies extra manipulation (extractions or precipitation). To avoid these extra steps, an *in situ* Fmoc removal strategy can be used. This approach, previously introduced by our group in SPPS, minimizes waste by eliminating the filtration and washing steps. In this method, piperidine is added directly to the coupling reaction mixture to achieve Fmoc deprotection.^28,29^

Excess piperidine (16 equiv relative to Cyclover) was added directly to the reaction mixture, and the reaction mixture was stirred for 30 min, with progress monitored by TLC and HPLC (see the Supporting Information). After completion, the mixture was neutralized with an aqueous solution of 0.1 N HCl, and the product (H-RinkAmide-Cyclover **1**) was precipitated by addition of a 5-fold excess of ACN (10 mL), relative to the reaction mixture solvent. The resulting precipitate was collected by centrifugation, the supernatant discarded, and the product washed twice with EtOAc (5-fold excess) to ensure efficient removal of byproducts (Scheme 1). ^31^P NMR also revealed that there are no T3P®-related byproducts (see the Supporting Information).^30^ The product was dried, and the structure was confirmed by ^1^H NMR (see the Supporting Information). H-RinkAmide-Cyclover **1** was obtained in 98% isolated yield. The solubility of **1** was assessed prior to its application in LPPS. It was found that **1** exhibited excellent solubility (0.13 M) in nonpolar solvents like toluene, anisole, *n*-hexane, cyclohexane, and *n*-pentane while being insoluble in polar (protic and aprotic) solvents like DMF, water, methanol, ethanol, acetonitrile acetone, and ethyl acetate.

**Scheme 1:**
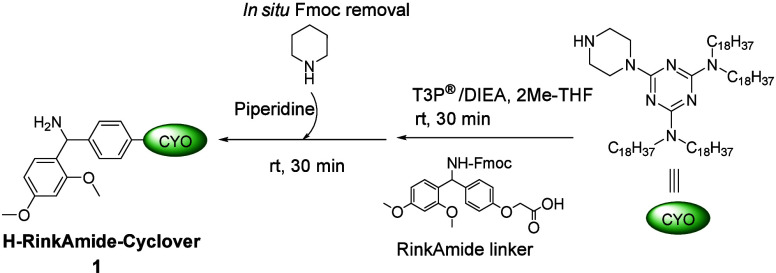
Synthesis of H-RinkAmide-Cyclover **1**

After the evaluation of the solubility, 2Me-THF^31^ and ACN/EtOAc were chosen as solvents for coupling and precipitation, respectively. As a proof of concept, model peptide Leu-enkephalin (H-YGGFL-NH_2_) was synthesized using H-RinkAmide-Cyclover **1** (0.067 mmol) following a three-step protocol of coupling, *in situ* Fmoc removal, and precipitation with 2Me-THF as the solvent of choice. Peptide elongation was carried out using 1.2 equiv of Fmoc-protected amino acids. Fmoc-Leu-OH (1.2 equiv) along with **1** (0.067 mmol, 1 equiv) was dissolved in 2Me-THF (2 mL), followed by the addition of DIEA as base to maintain a pH of ∼9. Subsequently, 4.0 equiv of a 50% solution of T3P® in EtOAc was added to the above solution. Fresh 2Me-THF contains traces of water/moisture, which increases to 10–12% (14 g/100 g at 20 °C) in due time. This increasing content of water in due course may eventually lead to partial hydrolysis of T3P®.^32,33^ Due to this, the initial 4.0 equiv was insufficient to achieve the complete reaction; therefore, additional T3P® (4.0 equiv) was added to offset this loss and ensure efficient complete coupling. The reaction pH was maintained at approximately 9 by addition of DIEA to facilitate effective coupling. The mixture was stirred at room temperature for 30 min until complete consumption of **1**, as confirmed by TLC and ninhydrin (see the Supporting Information).

Subsequent *in situ* Fmoc removal was performed by adding excess piperidine (16 equiv relative to Cyclover) directly to the reaction mixture and stirring for an additional 30 min. After the completion of the reaction, H-Leu-RinkAmide-Cyclover **2** was isolated as described above. The coupling–*in situ* Fmoc removal–precipitation cycle was repeated until H-Y­(*t*Bu)­GGFL-RinkAmide-Cyclover **3** was obtained. Each step was monitored by TLC and ninhydrin to confirm the complete conversion. The peptide was cleaved from the Cyclover tag by treatment with TFA/TIS/H_2_O (95:2.5:2.5; 1 mL) (100 mg/mL) for 2 h. After completion, the amount of TFA was reduced using a rotary evaporator, and cold *tert*-butyl methyl ether (TBME, 10-fold excess) was added to afford the desired peptide as a precipitate. The solid was collected by centrifugation, and the supernatant was decanted. The precipitate was washed twice with TBME to remove residual byproducts and the cleaved tag. ^31^P NMR also revealed that there are no T3P®-related byproducts (see the Supporting Information). The residue was vacuum-dried to obtain the desired Leu-enkephalin pentapeptide (**4**) via precipitation in 90% isolated yield (Scheme 2) over **1**. The crude purity and identity of the peptide were confirmed by RP-HPLC and LC-MS analysis. HPLC also indicated the presence of major impurities such as H-YGGL-NH_2_, H-YGFL-NH_2_, H-GGFL-NH_2_, and H-YGGGFL-NH_2_ (Figure 2A; see the Supporting Information).

**Figure 2:**
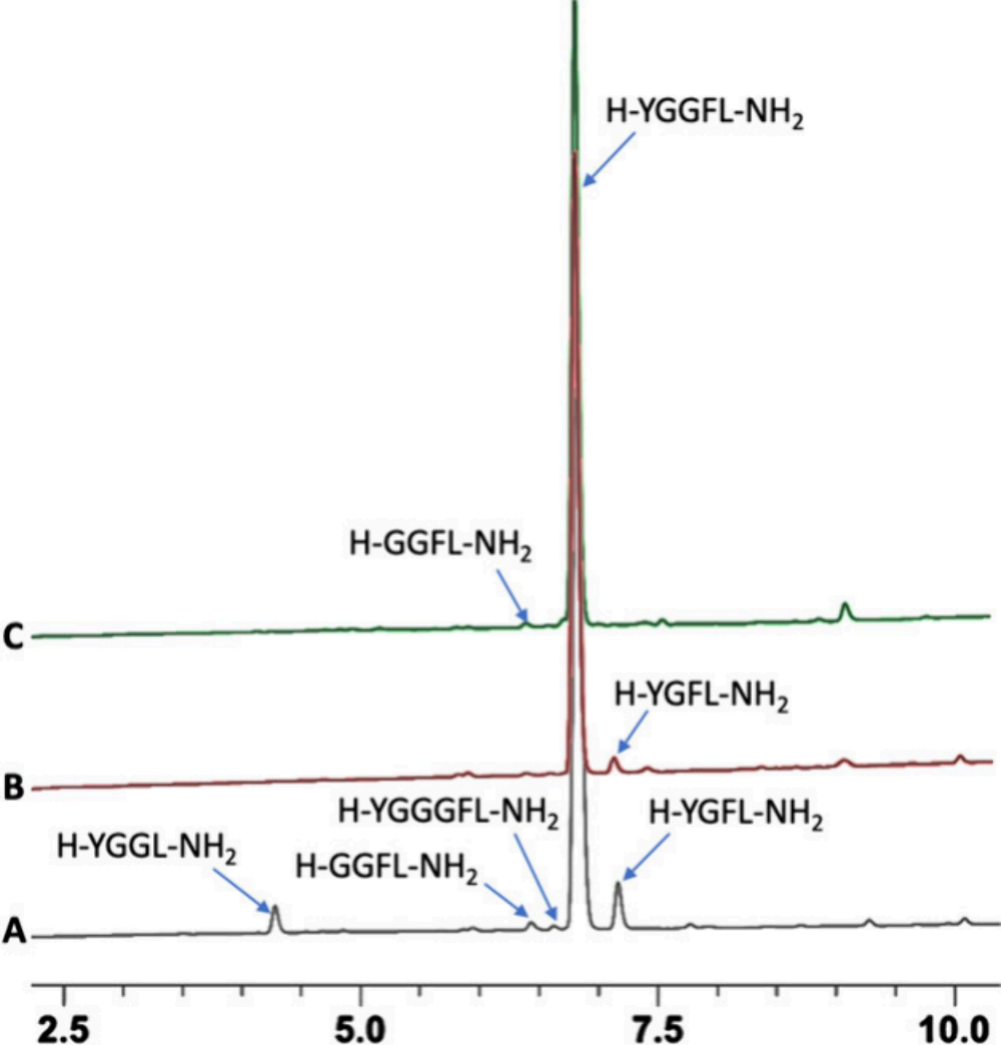
HPLC analysis of H-YGGFL-NH_2_ synthesized usingT3P®. (A) Synthesis in 2Me-THF with precipitation after each cycle of coupling and deprotection. (B) Synthesis in DCM with precipitation after each cycle of coupling and deprotection. (C) Synthesis in 2Me-THF with extraction after each cycle of coupling and deprotection. HPLC method: 5–60% B (ACN) into A (0.1% TFA in H_2_O) in 15 min at a flow rate of 1 mL/min at 220 nm.

**Scheme 2:**
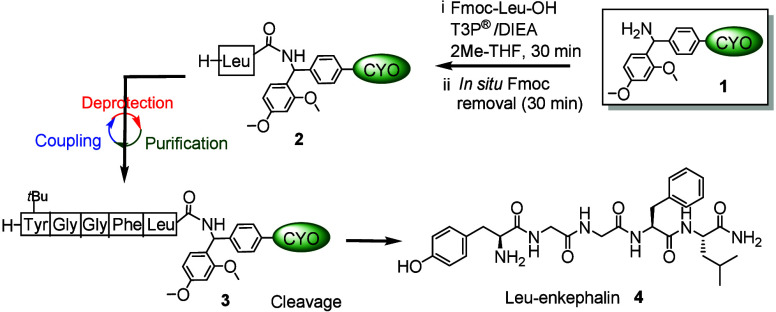
Schematic Representation of Leu-Enkephalin Peptide Synthesis Using a Cyclover-Assisted Liquid-Phase Strategy

The larger amount of T3P® required was thought to be necessary due to the moisture present in the solvent, which can degrade T3P® and reduce its effective coupling efficiency.^24,34^ Drying the solvent and performing the reaction in a closed system are other approaches to conveniently reduce the amount of moisture, thereby eliminating the hydrolysis of T3P®. However, these conditions will not be convenient for large-scale peptide synthesis. Moreover, during extraction, the solvent will be constantly extracted with water (three times in our case). This may again lead to wetting of the solvent, thereby hindering the next coupling process. Drying the solvent at each level will incur extra cost, and the process will be complicated to implement at the industrial scale. This extra amount of T3P® is due to the quenching in water, which was further validated by using DCM as the solvent (which is considered to absorb relatively less moisture compared to 2Me-THF). The same peptide was synthesized in nonhygroscopic DCM, reducing moisture uptake throughout the whole process The procedure used for the peptide synthesis was maintained exactly as explained above except that DCM was used as the solvent. It was found that the amount of T3P® required for the coupling was indeed smaller (6.0 equiv). Peptide H-YGGFL-NH_2_ synthesized in both 2Me-THF and DCM was obtained with comparable crude purity as shown in Figure 2B. Hence, the overall results demonstrate that 2Me-THF serves not only as a greener solvent but also as an effective reaction medium for LPPS, delivering performance comparable to that of ecologically less favorable DCM.

Considering the excess of solvents needed for precipitation, an identical synthesis of Leu-enkephalin (**4**) was repeated, wherein extraction was used as a process to isolate intermediates. The coupling reaction was performed in 2Me-THF (2 mL) using T3P® (8.0 equiv) and DIEA (16 equiv) while maintaining the pH around 9 as explained above. Upon completion of the reaction (as monitored by TLC and ninhydrin), an excess of piperidine (32 equiv) was added directly to the reaction mixture for *in situ* Fmoc deprotection. The reaction mixture was then stirred for additional 30 min to complete Fmoc removal. The increased excess amount of piperidine was added to facilitate complete formation of the unreactive dibenzofulvene (DBF) adduct with piperidine. Upon completion, the reaction mixture was then extracted with 0.1 N HCl (1-fold excess, relative to the reaction mixture solvent). The 2Me-THF layer was separated and further extracted with NaHCO_3_ and a brine solution, keeping 1-fold excess each. The 2Me-THF layer was then filtered by using anhydrous MgSO_4_. The organic layer then contained the required product (**2**) with an unreactive DBF–piperidine adduct (insoluble in the aqueous layer). It was then used directly for the next coupling. The coupling–*in situ* Fmoc removal–extraction cycle was repeated until the completion of the formation of H-Y­(*t*Bu)­GGFL-RinkAmide-Cyclover **3**, which was precipitated using excess ACN (10 mL, 5-fold) and EtOAc (10 mL, 5-fold) to ensure elimination of the DBF adduct from the product mixture. The peptide was then cleaved from the Cyclover tag using TFA/TIS/H_2_O (95:2.5:2.5) for 2 h, followed by precipitation with TBME as explained above for the precipitation approach. H-YGGFL-NH_2_ was obtained in 93% isolated yield (see the Supporting Information) using this extraction approach. The crude purity and identity of the peptide were determined and confirmed by HPLC and LC-MS analysis (Figure 2C). After comparing the precipitation (for both 2Me-THF and DCM) and extraction for Leu-enkephalin synthesis, the latter was found to consume less solvent (see below) with >98% purity compared to the other two syntheses, as shown in Figure 2.

To further support the sustainability of the extraction process, the PMI and cEF of Leu-enkephalin were calculated. PMI (and cEF) is defined as the total mass (including water) divided by the product obtained.^35^ In our cases, water waste has also been considered in the calculations of the green metrics. The precipitation method gave PMI and cEF values of 8426 and 8425, respectively. In the case of the extraction method, the PMI and cEF were significantly decreased to 3182 and 3181, respectively, clearly depicting an ∼2.7-fold decrease in the values. For comparison, previously reported SPPS with *in situ* Fmoc removal by our group exhibited PMI and cEF values of 571.0 and 570.0,^28^ respectively, whereas those of standard SPPS were found to be 2242.6 and 2241.6, respectively.^28^ Although the PMI and cEF of the current LPPS method are higher than those of SPPS, it remains unoptimized.^36^ The increased PMI primarily arises from the excessive use of solvents during precipitation/extraction steps with solvent consumption compounding across successive stages of the process. Careful optimization and fine-tuning of solvent volumes at each precipitation/extraction step are therefore critical to effectively reduce the overall PMI. The observed ∼2.7-fold reduction achieved by replacing precipitation with extraction underscores the potential for further improvements in these sustainability metrics.

After the successful synthesis of model peptide Leu-enkephalin **4**, synthesis of protected linear oxytocin H-C­(Acm)­YIQNC­(Acm)­PLG-NH_2_ **7** was attempted (Scheme 3). Oxytocin is a nonapeptide that contains two cysteine residues. The protected linear oxytocin was synthesized using Cyclover and T3P® as mentioned above to assess the effectiveness of the coupling reagent in LPPS.

**Scheme 3:**
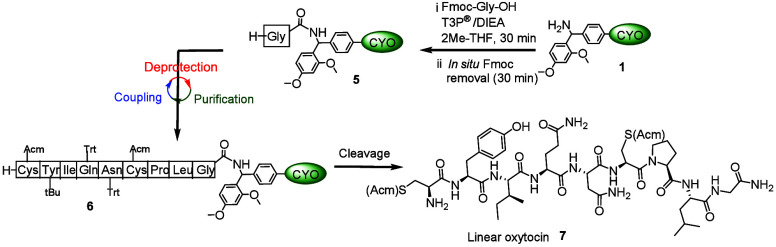
Schematic Representation of the Synthesis of Linear Oxytocin Using a Cyclover-Assisted Liquid-Phase Strategy

Two syntheses were performed again, comparing precipitation and extraction for peptide synthesis. In the first coupling step, Fmoc-Gly-OH (1.2 equiv) was attached to H-RinkAmide-Cyclover **1** (0.067 mmol) in 2Me-THF (2 mL) using T3P® (8.0 equiv) and DIEA (16 equiv) while maintaining a pH of ∼9. The reaction mixture was stirred at room temperature for 30 min until the reaction reached completion as confirmed by TLC and ninhydrin. *In situ* Fmoc removal was then performed by adding excess piperidine, and the reaction mixture was stirred for 30 min. In the case of precipitation, 16 equiv of piperidine was used, while in case of extraction, 32 equiv was used (again to ensure complete formation of the unreactive DBF–piperidine adduct). After Fmoc removal, the reaction mixture was neutralized by 0.1 N HCl. H-Gly-RinkAmide-Cyclover **5** was obtained in the case of precipitation and extraction as explained above. The coupling–*in situ* Fmoc removal–precipitation/extraction cycle was repeated until H-C­(Acm)­YIQNC­(Acm)­PLG-RinkAmide-Cyclover **6** was assembled. Each step was monitored by TLC and ninhydrin. In both cases, the peptides were cleaved from the tag using TFA/TIS/H_2_O (95:2.5:2.5, 100 mg/mL) for 2 h. After completion, the amount of TFA was reduced using a rotary evaporator followed by the addition of TBME (10-fold excess) to precipitate the desired peptide. Following centrifugation, the precipitate was collected and the supernatant was decanted. The precipitate was then washed twice with TBME and dried under a vacuum to obtain protected linear oxytocin. Isolation by either precipitation or extraction afforded the desired product in a similar isolated yield (86% or 87%, respectively; see the Supporting Information) and crude purity (>97% or >95% purity, respectively), as shown in Figure 3.

**Figure 3:**
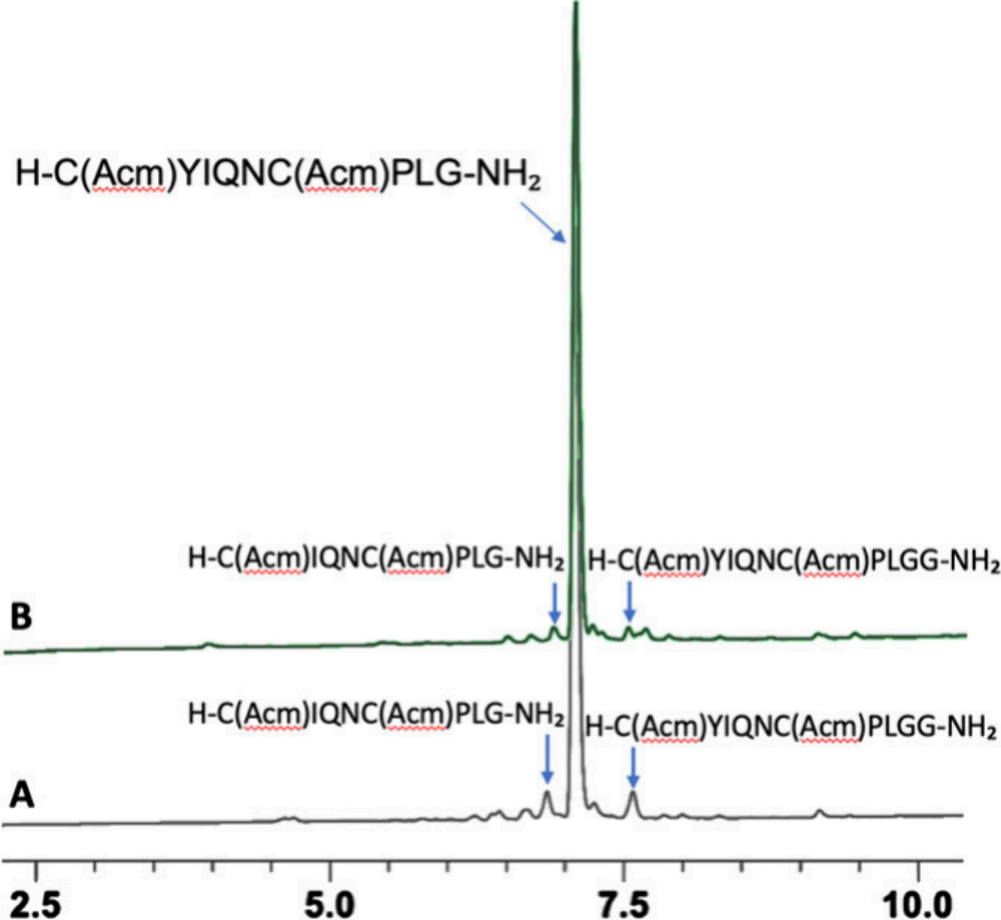
HPLC analysis of linear oxytocin synthesized with T3P®. (A) Synthesis in 2Me-THF with precipitation after each cycle of coupling and deprotection. (B) Synthesis in 2Me-THF with extraction after each cycle of coupling and deprotection. HPLC method: 0–60% B (ACN) into A (0.1% TFA in H_2_O) in 15 min at a flow rate of 1 mL/min at 220 nm.

In summary, Cyclover proved to be an effective tag for LPPS, enabling efficient peptide syntheses with both precipitation and extraction. When used in combination with T3P® as the coupling reagent, peptide elongation proceeded smoothly under mild reaction conditions. A key advantage of T3P® is the formation of a water-soluble byproduct, which can be easily removed by aqueous extraction, thus avoiding the difficult isolation steps often associated with other coupling reagents.

The tunable solubility of Cyclover allows homogeneous coupling in 2Me-THF, while product isolation can be achieved either by precipitation using excess ACN or by extraction through sequential washes with 0.1 N HCl, NaHCO_3_, and brine. Model peptide Leu-enkephalin was obtained in high isolated yield and high crude purity. PMI and cEF analyses further underscore the advantages of the extraction protocol (Table 1).

**Table 1:** Green Metrics for Leu-Enkephalin Synthesis via Precipitation and Extraction

Green Metric	Precipitation	Extraction
PMI	8426	3182
cEF	8425	3181

The extraction route showed a 2.7-fold reduction compared to that of precipitation. Similar purities were achieved for protected linear oxytocin using both isolation strategies.

Overall, Cyclover-assisted LPPS using T3P® as a peptide coupling reagent offers a sustainable and versatile platform for accessing complex peptides under mild and environmentally friendly conditions. The marked reduction in waste generation observed with the extraction method highlights the potential of Cyclover-based LPPS in combination with T3P® to deliver target peptides with excellent isolated yields and purity in a greener manner.

## Supplementary Material



## Data Availability

The data underlying this study are available in the published article and its Supporting Information.

## Notes

General information, experimental procedures, and HPLC spectra (PDF)
