# Supplementary material for: Cyclover-Assisted Liquid-Phase Peptide Synthesis Using T3P® as a Green Coupling Reagent
Source: Org Lett. 2026 Feb 24;28(9):3046–51. doi: 10.1021/acs.orglett.6c00353 (PMC12973291; doi:10.1021/acs.orglett.6c00353)
Supplement: Supplementary file 1 [file ol6c00353_si_001.pdf]

Cyclover-Assisted Liquid-Phase Peptide Synthesis Using T3P® as a Green Coupling Reagent

Priyanka Kushwaha,[1,*] Marvin Mantel,[2] Peter Talbiersky,[2] Yongfu Li,[3] Anamika Sharma,[1] Beatriz G. de la Torre,[1,4] Fernando Albericio[1,5,*]

[1]Peptide Science Laboratory, School of Chemistry and Physics, University of KwaZulu-Natal, Westville, Durban 4000, South Africa

[2]Biotide Core, LLC, 33815 SE Eastgate Circle, Corvallis, OR 97333, USA

[3]Curia Germany GmbH, Industriepark Höchst D569, 65926 Frankfurt am Main, Germany

[4]School of Laboratory Medicine and Medical Sciences, College of Health Sciences, University of KwaZulu-Natal, Durban 4041, South Africa

[5]Department of Inorganic and Organic Chemistry, University of Barcelona, 08026-Barcelona, Spain

Corresponding authors: Priyanka Kushwaha (priyankakushwaha877@gmail.com); Fernando Albericio (albericio@ukzn.ac.za)

Table of Contents

1 General Information
2 Experimental Procedure
3 Cyclover Tag-Assisted Liquid-Phase Peptide Synthesis (LPPS)
4 Attachment of RinkAmide Linker to Cyclover Tag and *in-situ* Fmoc Cleavage
5 General Procedure for LPPS *via* the Precipitation Approach
6 General Procedure for LPPS *via* the Extraction Approach
7 General Procedure for Cleavage of Peptides from the Cyclover Tag
8 Synthesis of H-YGGFL-NH_2 (**4**) *via* the Precipitation Approach
9 Global Deprotection and Isolation of H-YGGFL-NH_2 from Cyclover
10 Synthesis of H-Y(*t*Bu)GGFL-RinkAmide-Cyclover using 6.0 eq. of T3P® and DCM
11 Synthesis using Extraction Approach
12 Global Deprotection and Isolation of H-YGGFL-NH_2 (**4**) from Cyclover
13 Synthesis of Oxytocin H-C(Acm)YIQNC(Acm)PLG-NH_2 (**7**) *via* Precipitation / Extraction Approach

General Information

All reagents and solvents were purchased from commercial suppliers and used as received without further purification. Fmoc amino acids were purchased from Chempure (Bangalore India). Cyclover amine was purchased from Biotide. T3P® (50% solutions) were provided by Curia Germany GmbH. Organic solvents, dichloromethane (DCM), 2Me-THF, EtOAc and HPLC quality acetonitrile (ACN), were purchased from Merck. Trifluoracetic acid (TFA), Triisopropylsilane (TIS) and 0.1N HCl was purchased from Merck. Milli-Q water was used for RP-HPLC. Analytical HPLC was performed Shimadzu using a Phenomenex AerisTMC18 (3.6 µm, 4.6 × 150 mm) column, with a flow rate of 1.0 mL/min and UV detection at 220 nm. Lab solution software was used for data processing. Buffer A: 0.1% TFA in H_2O; buffer B: 0.1% TFA in ACN. LCMS was performed on a Thermo Fisher Scientific UltiMate 3000 UHPLC-ISQTM EC single quadrupole mass spectrometer in positive ion mode using a Phenomenex AerisTM C18 (3.6 µm, 4.6 × 150 mm) column. Buffer A: 0.1% formic acid in H_2O; buffer B: 0.1% formic acid in ACN. Analytical HPLC for Cyclover tagged peptides were performed on an Agilent 1100 system using a Phenomenex Luna 5µmC5 100A° (5µm, 4.6 × 50 mm) column, with flow rate of 1.0 mL/min and UV detection at 254 nm. Chemstation software was used for data processing. NMR spectra (^{1}H NMR) were recorded on a Bruker AVANCE III 600 MHz spectrometer. Chemical shift values are expressed in parts per million (ppm).

HPLC method A: 5-60% B (ACN) into A (0.1% TFA in H_2O) in 15 min with flowrate 1 mL/min at 220 nm (for H-YGGFL-NH_2).

HPLC method B: 0-60% B (ACN) into A (0.1% TFA in H_2O) in 15 min with flowrate 1 mL/min at 220 nm (for linear oxytocin) H-C(Acm)YIQNC(Acm)PLG-NH_2.

HPLC method C: 50-100% B (THF) into A (0.1% TFA in H_2O) in 15 min with flowrate 1 mL/min at 254 nm (for all Cyclover-tagged peptide).

Experimental Procedure

Cyclover Tag-Assisted Liquid-Phase Peptide Synthesis:

All peptides were assembled in a 50 mL falcon tube, with Fmoc/*t*Bu methodology using T3P®/DIEA as coupling agents. Fmoc removal was carried *in-situ* i.e., piperidine was added to the Cyclover tagged peptide without precipitation/extraction of coupling cocktail.

Attachment of RinkAmide Linker to Cyclover Tag and *in-situ* Fmoc Cleavage[1,2]:

Cyclover amine (500 mg, 0.415 mmol, 1.0 eq.) was dissolved in 2Me-THF (5 mL, 100 mg/mL). To this solution, Rink amide linker (270 mg, 0.5 mmol, 1.2 eq.) and T3P® (50% in EtOAc, 830 µL, 1.7 mmol, 4.0 eq.) were added sequentially at room temperature, pH of the reaction balanced up to ~9 using DIEA (580 µL, 3.32 mmol, 8.0 eq.).[3] The reaction mixture was stirred for 30 min and monitored by TLC (*n*-hexane:EtOAc = 7:3) and HPLC. TLC was performed by withdrawing 10 µL of the reaction mixture in an eppendorf, then ACN was added to precipitate the product of interest, upon centrifugation the precipitate was used for TLC and HPLC analysis (The same protocol was followed throughout unless otherwise specified) (**Figure 1-4**). After completion, piperidine (656 µL, 6.64 mmol, 16.0 eq.) was added and the mixture was stirred for 30 min. The reaction was then neutralized with 0.1 N HCl (4 mL). After 2 min, acetonitrile (ACN) (50 mL) was added to precipitate the product. The mixture was centrifuged, and the supernatant was decanted. The precipitate was washed with ethyl acetate (EtOAc) (3 × 50 mL) and dried in *vacuo* to give **H-RinkAmide-Cyclover** as a white solid (709 mg, 98% yield).

^{1}H NMR of Fmoc-RinkAmide-Cyclover: (600 MHz, $CDCl_3$): δ 7.30 (d, *J* = 9Hz, 2H; H_{Ar}), 7.11 (d, *J* = 7.8Hz, 1H; H_{Ar}), 6.89 (d, *J* = 7.8Hz, 2H; H_{Ar}), 6.42 (2H; H_{Ar}), 5.38 (s, 1H, NCH), 4.69 (s, 2H, OCH_2), 3.74 (s, 3H; O-CH_3), 3.72 (s, 3H; O-CH_3), 3.73-3.70 (m, 2H, NCH_2(Piperazine)), 3.64-3.59 (m, 2H, NCH_2(Piperazine)), 3.60-3.52 (m, 2H, NCH_2(Piperazine)), 3.45-3.38 (m, 8H, NCH_2(Piperazine) and NCH_2(Triazine)), 1.72-1.68 (m, 7H, CH_2(aliphatic chain)), 1.54 (CH_2(aliphatic chain) and solvent peak), 1.28-1.22 (m, 123H, CH_2(aliphatic chain)), 0.89-0.84 (m, 12H, CH_3) ppm.

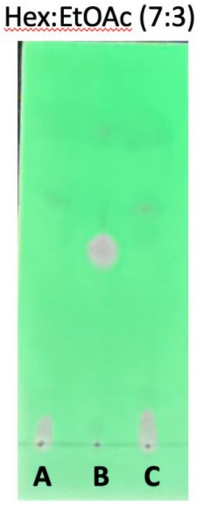

A – Cyclover amine (CYO)
B –Fmoc-RinkAmide-CYO
C - H-RinkAmide-CYO

Figure 1. TLC analysis of **A)** Cyclover(CYO), **B)** Fmoc-RinkAmide-CYO, and **C)** H-RinkAmide-CYO

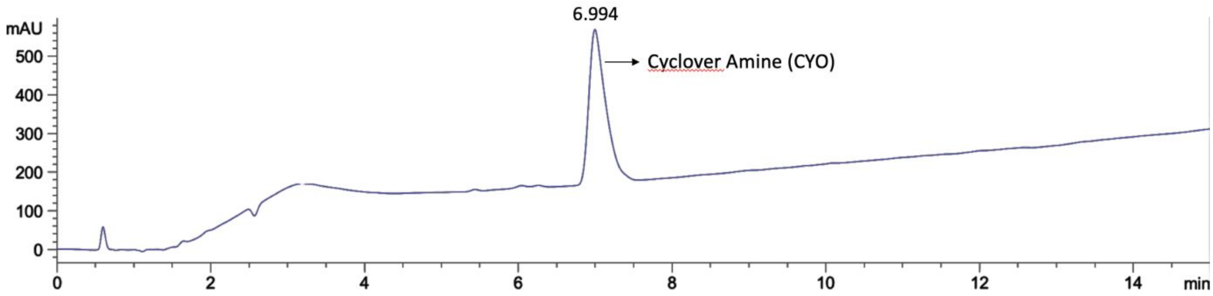

Figure 2. HPLC analysis of Cyclover (CYO) using HPLC Method C (50-100% B (THF) into A (0.1% TFA in H_2O) in 15 min with flowrate 1 mL/min at 254 nm)

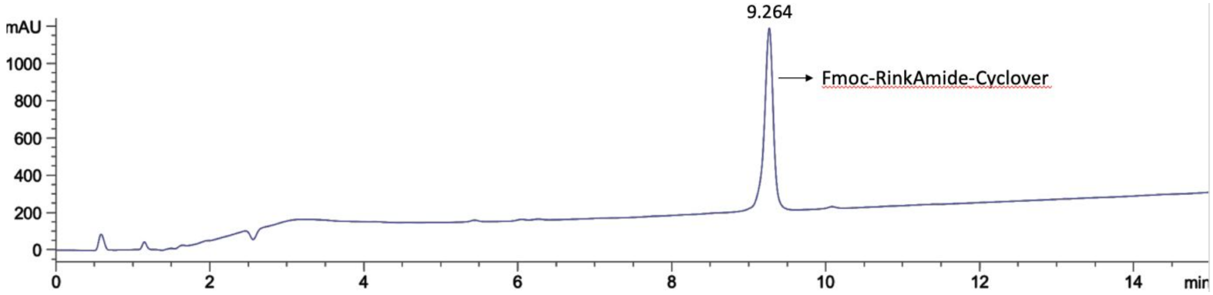

Figure 3. HPLC analysis of Fmoc-RinkAmide-Cyclover using HPLC Method C (50-100% B (THF) into A (0.1% TFA in H_2O) in 15 min with flowrate 1 mL/min at 254 nm).

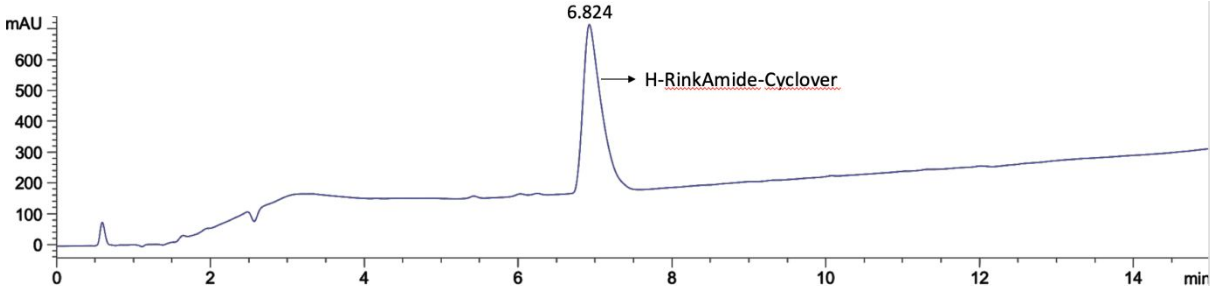

Figure 4. HPLC analysis of H-RinkAmide-Cyclover using HPLC Method C (50-100% B (THF) into A (0.1% TFA in H_2O) in 15 min with flowrate 1 mL/min at 254 nm).

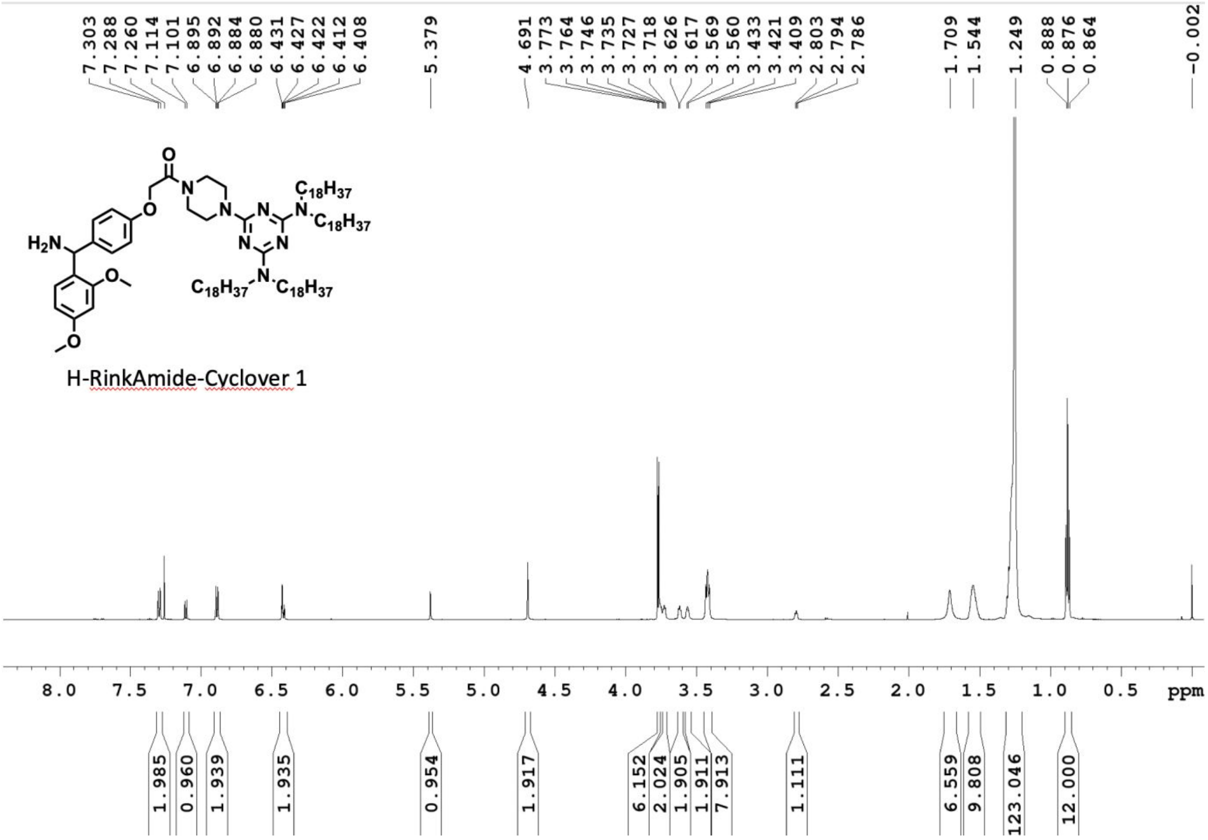

Figure 5. ^{1}H NMR of H-RinkAmide-Cyclover in $CDCl_3$

General Procedure for Liquid Phase Peptide Synthesis (LPPS) *via* the Precipitation Approach:

Fmoc–AA–OH (1.2 eq.), T3P® (50% in EtOAc, 8.0 eq.), and DIEA (16 eq.) were added to a solution of H–RinkAmide-Cyclover in 2-MeTHF (100 mg/2mL). The reaction mixture was stirred at room temperature for 30 min. Coupling progress was monitored by TLC as explained above (*n*-hexane:EtOAc = 7:3). After completion, piperidine (16.0 eq. relative to starting material Cyclover) was added directly to the reaction mixture to remove the Fmoc group. The mixture was stirred at room temperature for 30 min. Deprotection was confirmed by TLC and HPLC, indicating formation of the H-AA_1-RinkAmide-Cyclover. After the reaction was completed, the mixture was neutralized with 0.1 N HCl (1-fold with respect to reaction mixture) and stirred for 2 min. ACN (10 mL, 5-fold excess relative to reaction mixture) was then added to precipitate. The mixture was centrifuged, and the supernatant was removed. The precipitate was washed three times with EtOAc (10 mL each, 5-fold excess relative to reaction mixture) and centrifuged. After centrifugation, supernatant was decanted. The final precipitate was dried in *vacuo* to afford H-AA_1-RinkAmide-Cyclover as a white solid.

The synthesis of peptide on the Cyclover tag was carried out by iterative coupling and *in-situ* Fmoc deprotection cycles. Completion of each coupling and deprotection step was monitored by TLC (where *n*-hexane:EtOAc mobile phase was varied from 7:3 to 1:1) and HPLC. After each cycle, the Cyclover-tagged peptide was precipitated, following the general procedure described above. These steps were repeated sequentially until the final peptide was obtained.

General Procedure for Liquid Phase Peptide Synthesis *via* the Extraction Approach:

Fmoc-AA-OH (1.2 eq.), T3P® (50% in EtOAc, 8.0 eq.), and DIEA (16.0 eq.) were added to a solution of H-RinkAmide-Cyclover in 2-MeTHF (100 mg/2mL). The reaction mixture was stirred at room temperature for 30 min. Coupling progress was monitored by TLC (*n*-hexane:EtOAc = 7:3). After completion, piperidine (32.0 eq. relative to starting material) was added directly to the reaction mixture to remove the Fmoc group. The mixture was stirred at room temperature for 30 min. Deprotection was confirmed by TLC, indicating formation of the H-AA_1-RinkAmide-Cyclover (**Figure 6**). After completion of the reaction, the reaction mixture was transferred to a 50 mL separating funnel and neutralized with 0.1 N HCl (1-fold, relative to starting material). The mixture was shaken gently and allowed to stand until the layers separated. The organic layer containing the product was collected, while water-soluble impurities remained in the aqueous phase. The organic layer was then washed sequentially with saturated $NaHCO_3$ (1-fold, relative to starting material) solution and with brine (1-fold, relative to starting material). The washed organic layer was dried over anhydrous $MgSO_4$ (0.5 g/2mL), filtered, and used directly for the next coupling step. TLC analysis of organic layer showed the bottom spot of H-AA_1-RinkAmide-Cyclover and DBF-piperidine adduct spot is showing high retention factor (R_f) value than H-AA_1-RinkAmide-Cyclover. DBF-piperidine adduct is a stable molecule which does not hamper during coupling and deprotection and it is not soluble in water. The synthesis of peptide on the Cyclover tag was carried out by iterative coupling and *in-situ* Fmoc deprotection cycles. After each cycle, the Cyclover-tagged peptide was extracted, following the procedure described above. These steps were repeated sequentially until the final peptide was obtained.

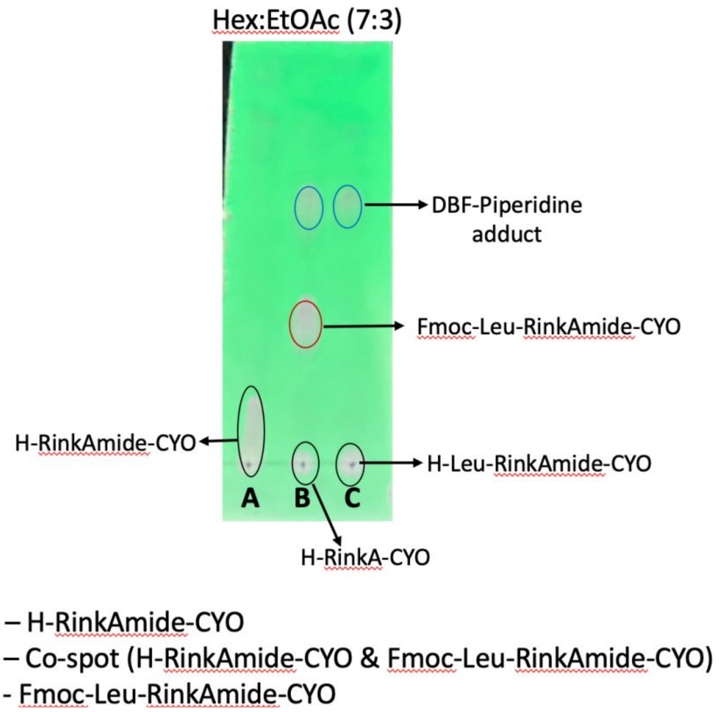

Figure 6. TLC analysis of Fmoc-L-RinkAmide-Cyclover *via* extraction method: **A)** H-RinkAmide-CYO, **B)** Co-spot of H-RinkAmide-CYO and Fmoc-Leu-RinkAmide-CYO using 8.0 eq. T3P®, **C)** H-Leu-RinkAmide-CYO

General Procedure for Cleavage of Peptides from the Cyclover Tag:

The Cyclover-tagged peptide was treated with cleavage cocktail (100 mg/mL) containing TFA:TIS:H_2O (95.0:2.5:2.5). The mixture was stirred at room temperature for 2 h. After completion, TFA amount was reduced. Peptide precipitation was induced by adding cold *tert*-butyl methyl ether (TBME) (10-fold excess relative to reaction mixture). The mixture was centrifuged, and the supernatant was decanted. The precipitate was washed and centrifuged twice with TBME (10-fold excess relative to reaction mixture) and dried in *vacuo*. The crude peptide (1 mg/mL) was dissolved in water and analysed by HPLC.

Experimental Procedure:

Scheme 1. Synthesis of H-YGGFL-NH$_2$ (4) *via* the Precipitation Approach

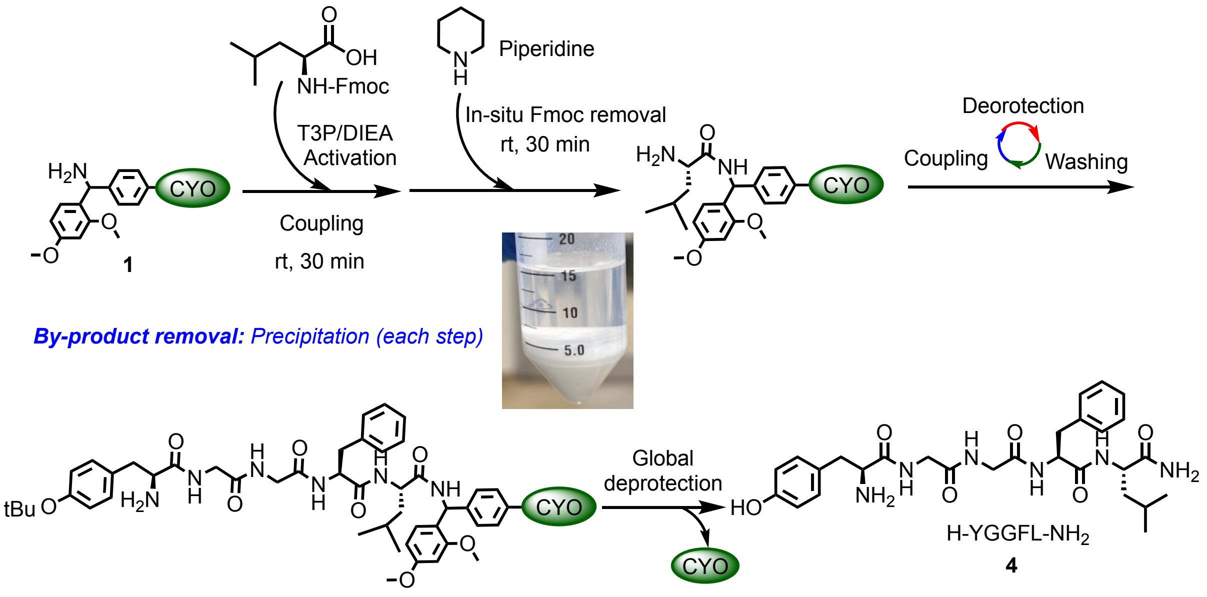

Attachment of Fmoc-Leu-OH to H-RinkAmide-Cyclover Using 4.0 eq. of T3P® and 2Me-THF:

The H-RinkAmide-Cyclover **1** (100 mg, 0.067 mmol, 1.0 eq.) was dissolved in 2-MeTHF (2 mL). To this, Fmoc-Leu-OH (29 mg, 0.08 mmol, 1.2 eq.), T3P® (50% in EtOAc, 134 µL, 0.27 mmol, 4.0 eq.), and DIEA (94 µL, 0.54 mmol, 8.0 eq.) were added. The mixture was stirred at room temperature for 30 min at room temperature. 10 µL of the reaction mixture was taken in an eppendorf, precipitated with ACN, centrifuged and the precipitate was used for TLC analysis (*n*-hexane/EtOAc = 7:3). As incomplete conversion was observed (**Figure 7**), additional T3P® (50% in EtOAc, 67 µL, 0.134 mmol, 2.0 eq.) and DIEA (47 µL, 0.27 mmol, 4.0 eq.) were added, and stirring was continued for an additional 30 min. Reaction progress was again monitored by TLC using the same precipitation-based method (**Figure 7**).

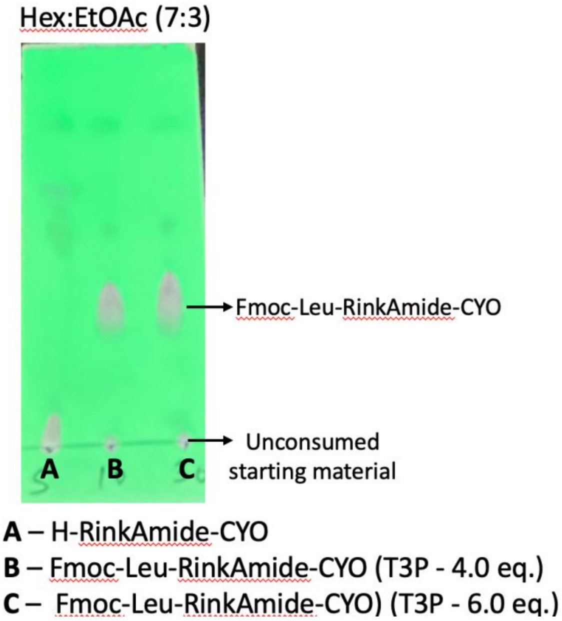

Figure 7. TLC analysis of Fmoc-L-RinkAmide-Cyclover: **A)** H-RinkAmide-CYO, **B)** Fmoc-Leu-RinkAmide-CYO using 4.0 eq. T3P®, **C)** Fmoc-Leu-RinkAmide-CYO with 6.0 eq. of T3P®.

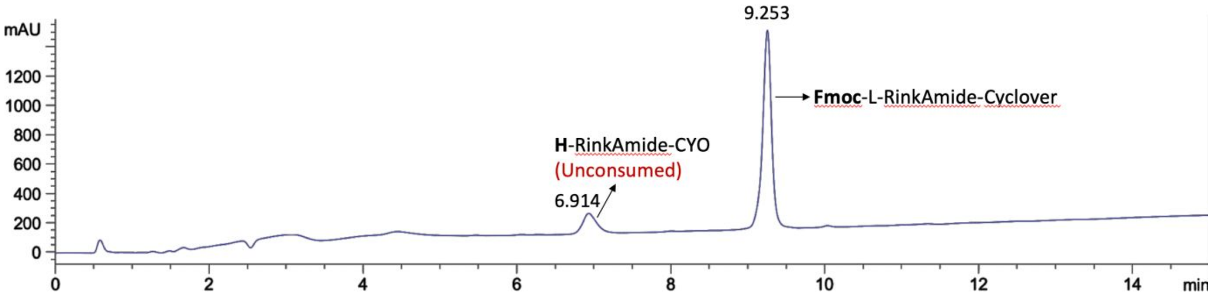

Figure 8. HPLC analysis of Fmoc-L-RinkAmide-Cyclover using 6.0 eq. of T3P® using HPLC Method C (50-100% B (THF) into A (0.1% TFA in H_2O) in 15 min with flowrate 1 mL/min at 254 nm).

Synthesis of H-Y(*t*Bu)GGFL-RinkAmide-Cyclover Using 8.0 eq. of T3P® and 2Me-THF:

H-RinkAmide-Cyclover (100 mg, 0.067 mmol, 1.0 eq.) was dissolved in 2-MeTHF (2 mL). Fmoc-Leu-OH (29 mg, 0.080 mmol, 1.2 eq.), T3P® (50% in EtOAc, 268 μL, 0.54 mmol, 8.0 eq.), and DIEA (187 μL, 1.07 mmol, 16.0 eq.) were added, and the reaction mixture was stirred at room temperature for 30 min. Reaction completion was monitored by TLC (*n*-hexane/EtOAc = 7:3) as explained above. After completion of the coupling, piperidine (106 μL, 1.07 mmol, 16.0 eq.) was added directly to the reaction mixture for *in-situ* Fmoc deprotection. The mixture

was stirred at room temperature for 30 min, and deprotection was confirmed by TLC (*n*-hexane/EtOAc = 7:3) (**Figure 9**). After completion of the reaction, as confirmed by TLC and HPLC, 0.1N HCl (1 mL) added to neutralize the reaction mixture. ACN (10 mL) was added to the reaction mixture to induce precipitation. The mixture was sonicated and then centrifuged, and the supernatant was discarded. The resulting precipitate was washed with EtOAc (10 mL), sonicated, and centrifuged. Supernatant was decanted. This EtOAc (10 mL) washing step was repeated twice to obtain the H-L-RinkAmide-Cyclover. Subsequently, chain elongation was continued by sequential coupling of the Fmoc-Phe-OH (32 mg, 0.08 mmol, 1.2 eq.) Fmoc-Gly-OH (24 mg, 0.08 mmol, 1.2 eq.) Fmoc-Gly-OH (24 mg, 0.08 mmol, 1.2 eq.) Fmoc-Tyr(*t*Bu)-OH (37 mg, 0.08 mmol, 1.2 eq.), T3P® (50% in EtOAc, 268 μL, 0.54 mmol, 8.0 eq.), and DIEA (187 μL, 1.07 mmol, 16.0 eq.). After each coupling step piperidine (106 μL, 1.07 mmol, 16.0 eq.) was added directly to the reaction mixture for *in-situ* Fmoc deprotection. After each *in-situ* Fmoc removal step Cyclover-tagged peptide was isolated by precipitation with ACN (10 mL) and EtOAc (10 mL). The final product was dried under *vacuo* to afford H-Y(*t*Bu)GGFL-RinkAmide-Cyclover as a white solid (125 mg, 92% yield).

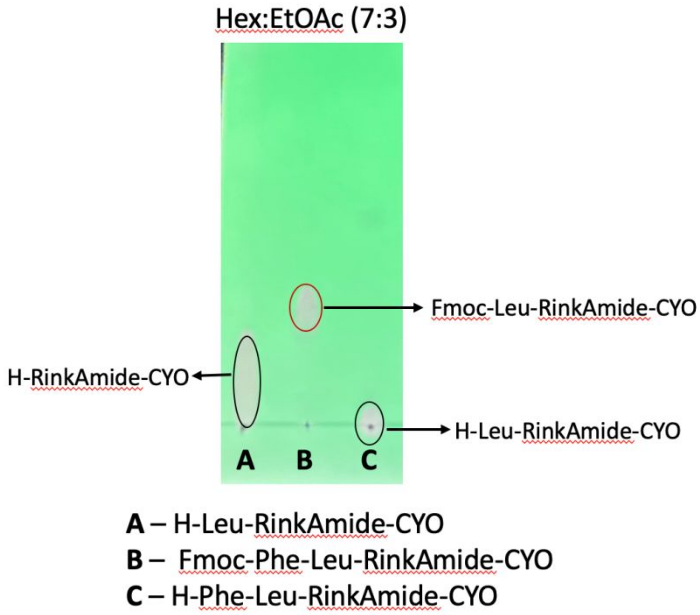

Figure 9. TLC analysis of: **A)** H-Leu-RinkAmide-CYO, **B)** Fmoc-Phe-Leu-RinkAmide-CYO using 8.0 eq. T3P®, **C)** H-Phe-Leu-RinkAmide-CYO.

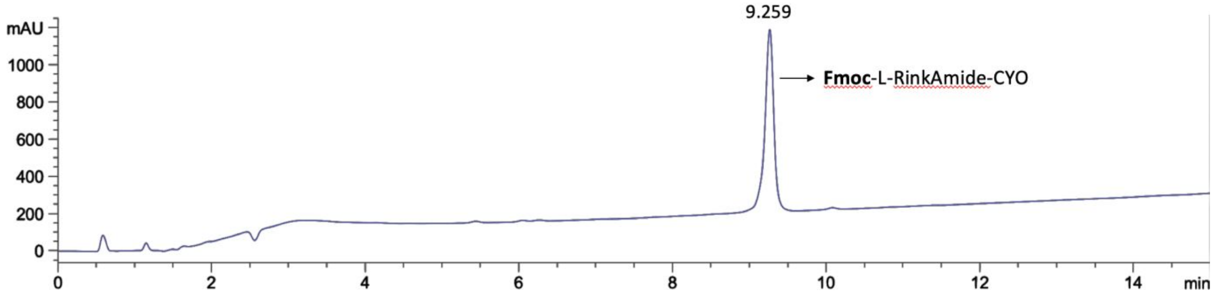

Figure 10. HPLC analysis of Fmoc-L-RinkAmide-Cyclover using 8.0 eq. of T3P® using HPLC Method C (50-100% B (THF) into A (0.1% TFA in H_2O) in 15 min with flowrate 1 mL/min at 254 nm).

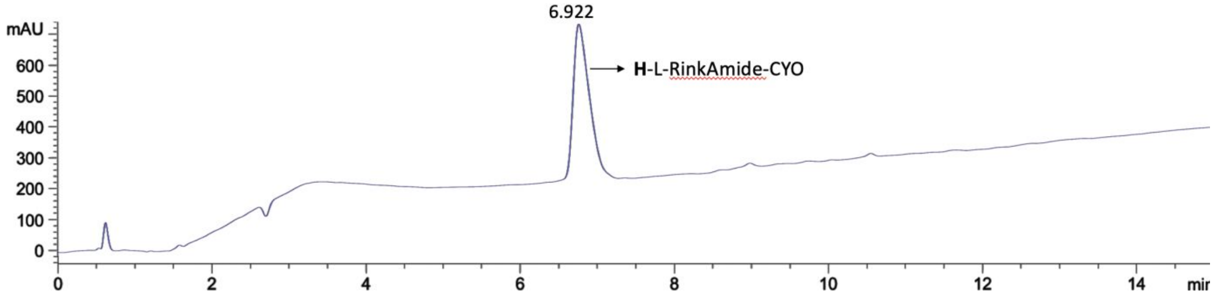

Figure 11. HPLC analysis of H-L-RinkAmide-Cyclover using HPLC Method C (50-100% B (THF) into A (0.1% TFA in H_2O) in 15 min with flowrate 1 mL/min at 254 nm).

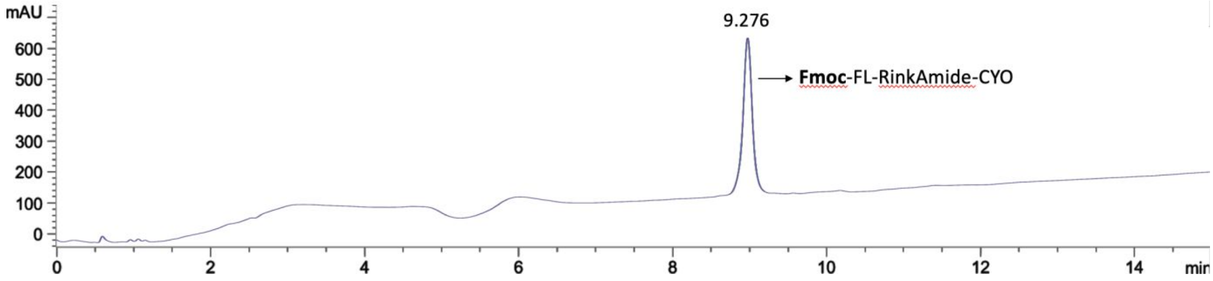

Figure 12. HPLC analysis of Fmoc-FL-RinkAmide-Cyclover using 8.0 eq. of T3P® using HPLC Method C (50-100% B (THF) into A (0.1% TFA in H_2O) in 15 min with flowrate 1 mL/min at 254 nm).

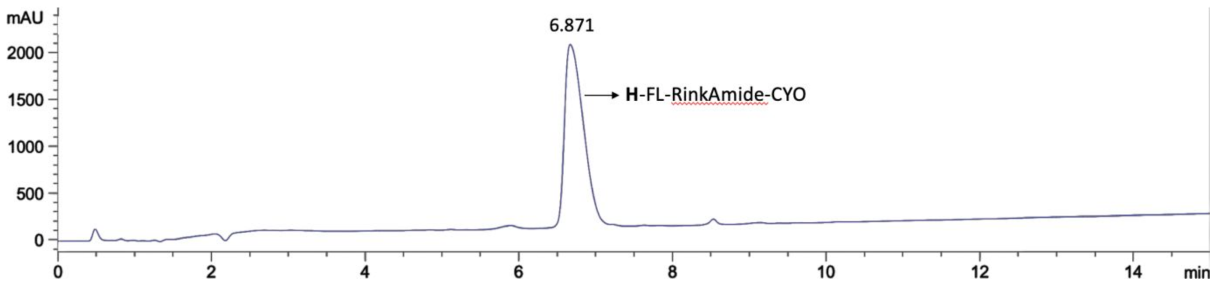

Figure 13. HPLC analysis of H-FL-RinkAmide-Cyclover using HPLC Method C (50-100% B (THF) into A (0.1% TFA in H_2O) in 15 min with flowrate 1 mL/min at 254 nm).

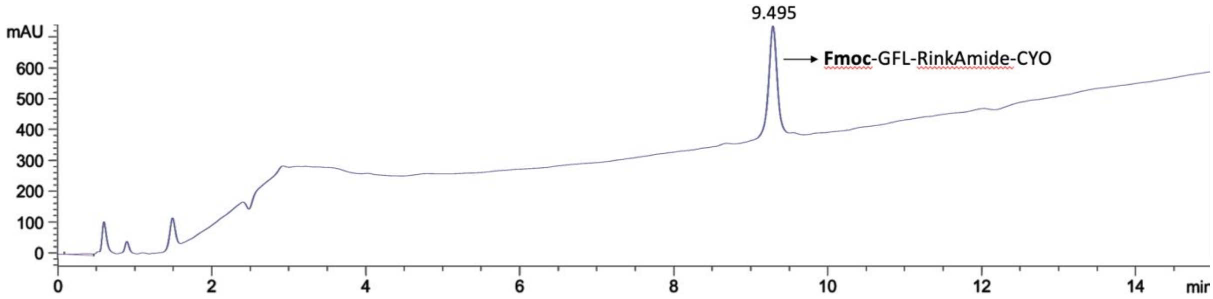

Figure 14. HPLC analysis of Fmoc-GFL-RinkAmide-Cyclover using 8.0 eq. of T3P® using HPLC Method C (50-100% B (THF) into A (0.1% TFA in H_2O) in 15 min with flowrate 1 mL/min at 254 nm).

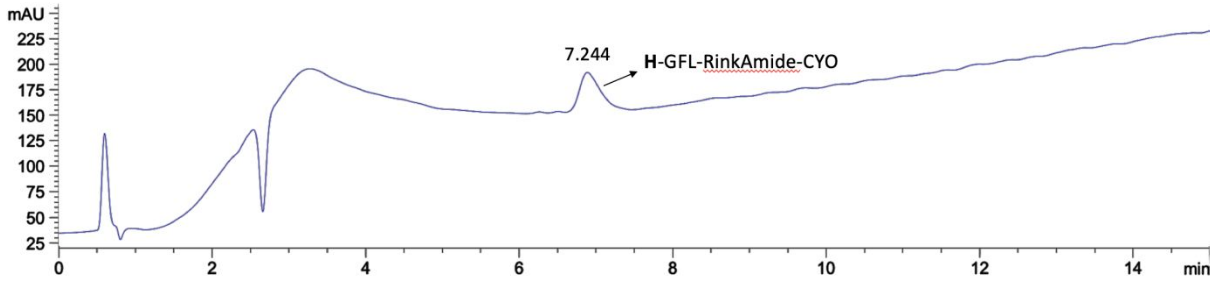

Figure 15. HPLC analysis of H-GFL-RinkAmide-Cyclover using HPLC Method C (50-100% B (THF) into A (0.1% TFA in H_2O) in 15 min with flowrate 1 mL/min at 254 nm).

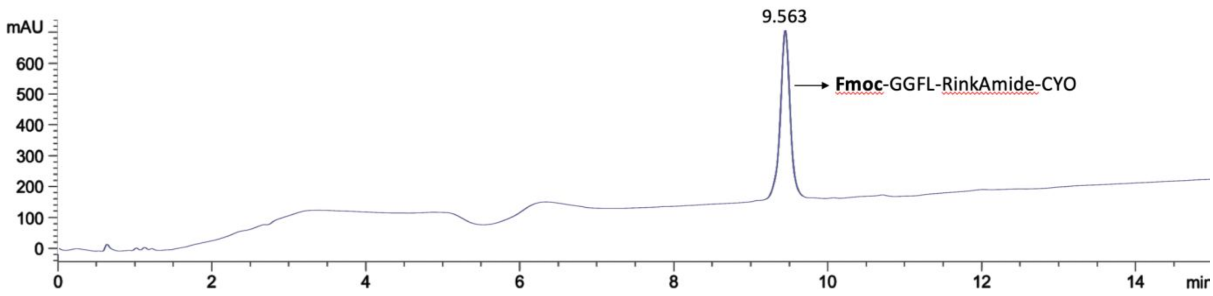

Figure 16. HPLC analysis of Fmoc-GGFL-RinkAmide-Cyclover using 8.0 eq. of T3P® using HPLC Method C (50-100% B (THF) into A (0.1% TFA in H_2O) in 15 min with flowrate 1 mL/min at 254 nm).

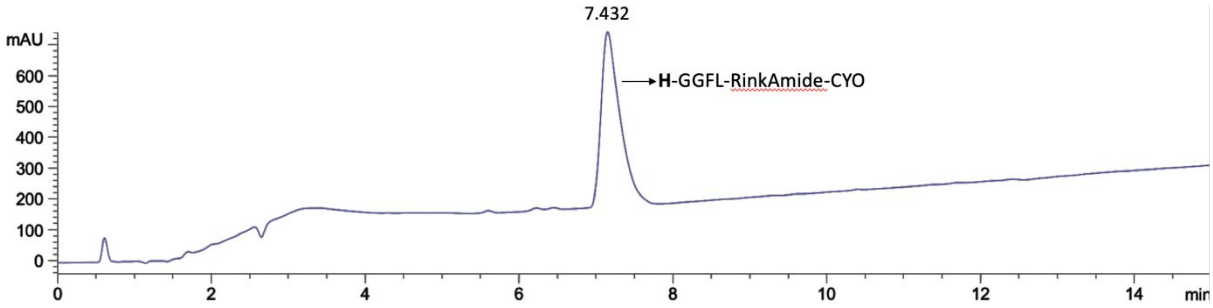

Figure 17. HPLC analysis of H-GGFL-RinkAmide-Cyclover using HPLC Method C (50-100% B (THF) into A (0.1% TFA in H_2O) in 15 min with flowrate 1 mL/min at 254 nm).

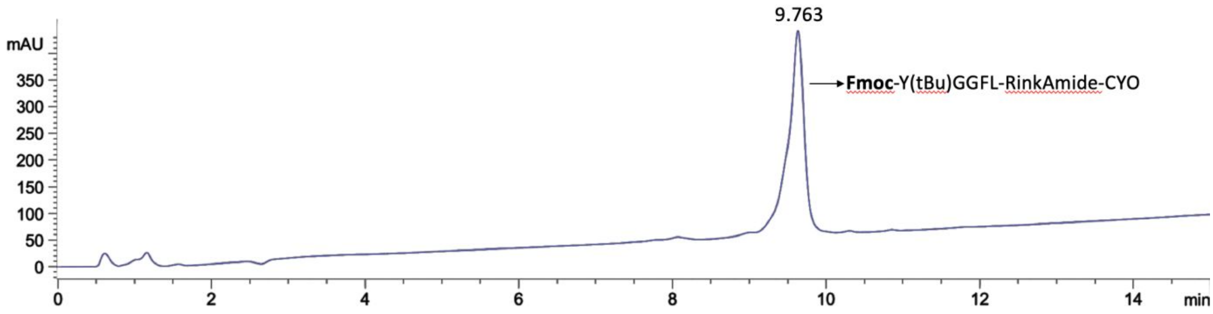

Figure 18. HPLC analysis of Fmoc-Y(*t*Bu)GGFL-RinkAmide-Cyclover using 8.0 eq. of T3P® using HPLC Method C (50-100% B (THF) into A (0.1% TFA in H_2O) in 15 min with flowrate 1 mL/min at 254 nm).

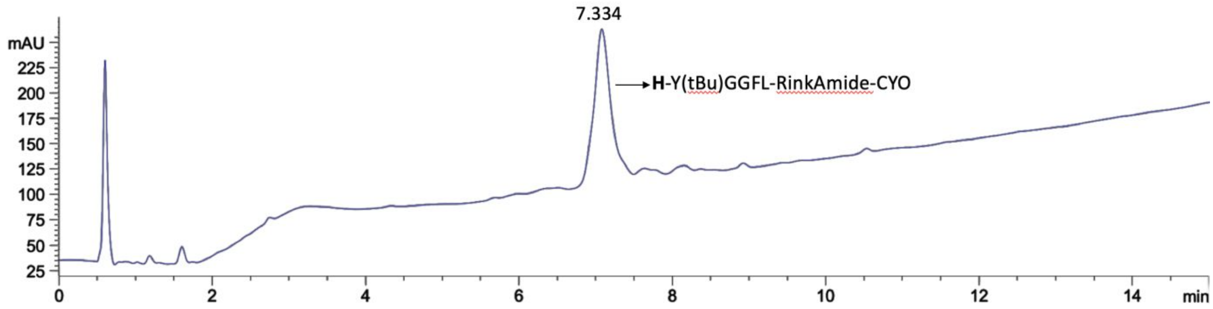

Figure 19. HPLC analysis of H-Y(*t*Bu)GGFL-RinkAmide-Cyclover using HPLC Method C (50-100% B (THF) into A (0.1% TFA in H_2O) in 15 min with flowrate 1 mL/min at 254 nm).

Global Deprotection and Isolation of H-YGGFL-NH_2 from Cyclover:

H-Y(*t*Bu)GGFL-RinkAmide-Cyclover (125 mg) was treated with a cleavage cocktail TFA/H_2O/TIS (95:2.5:2.5; 1 mL). The reaction mixture was stirred at room temperature for 2 h. After completion, TFA amount was reduced. Peptide precipitation was induced by the addition of cold TBME (10 mL, 10-fold excess to reaction mixture). The mixture was centrifuged, and the supernatant was decanted. The resulting precipitate was washed twice with cold TBME (10 mL), centrifuged, and dried under *vacuo* to get crude **H-YGGFL-NH_2** in (33 mg, 90%). The crude peptide was dissolved in water (1 mg/mL) and analysed by HPLC.

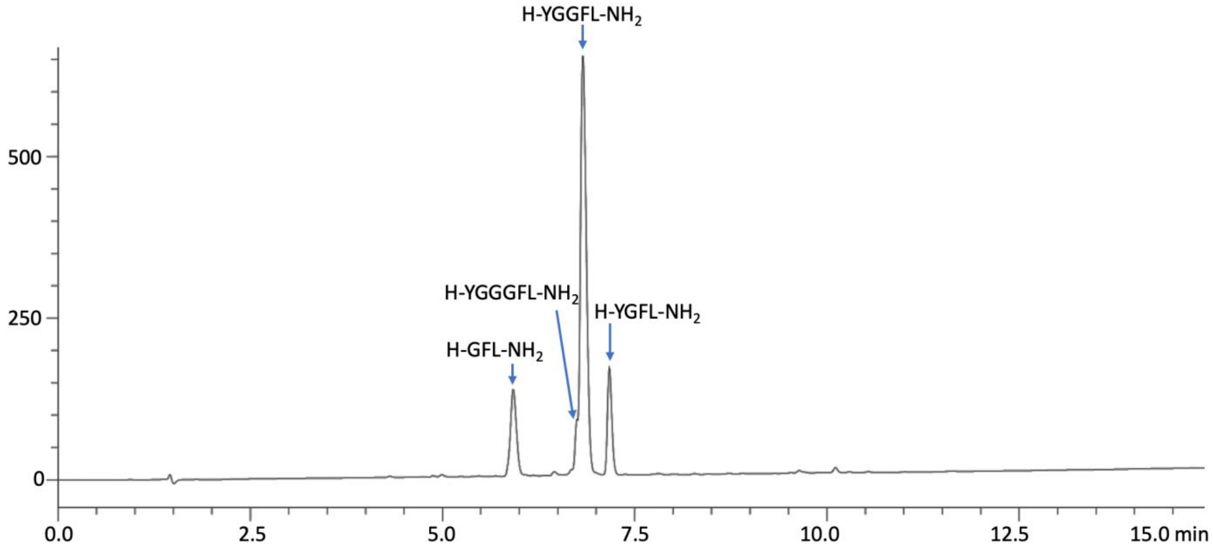

Figure 20. HPLC analysis (using HPLC Method A: 5-60% B (ACN) into A (0.1% TFA in H_2O) in 15 min with flowrate 1 mL/min at 220 nm) of the product obtained by LPPS of Leu-Enkephalin pentapeptide (H-YGGFL-NH_2) as using Fmoc-AA-OH: T3P®: [1.2:4.0 eq.], pH~9 with DIEA, 30 min in 2Me-THF. *In-situ* Fmoc removal (with 16.0 eq. piperidine, 30 min). Precipitation after Fmoc removal with ACN (1x10 mL) and EtOAc (3x10 mL).

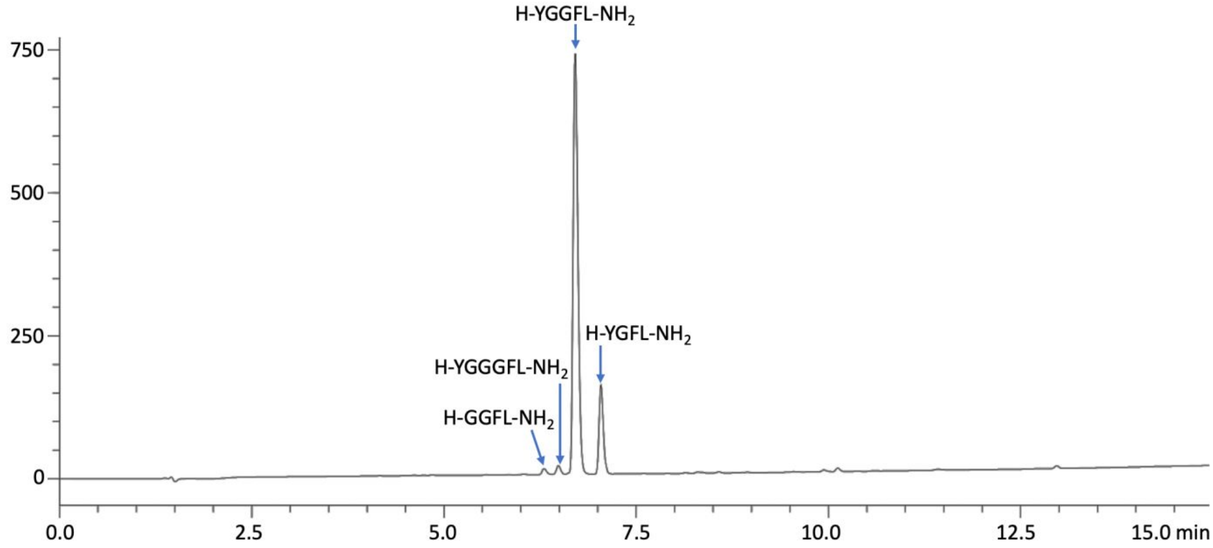

Figure 21. HPLC analysis (using HPLC Method A: 5-60% B (ACN) into A (0.1% TFA in H_2O) in 15 min with flowrate 1 mL/min at 220 nm) of the product obtained by LPPS of Leu-Enkephalin pentapeptide (H-YGGFL-NH_2) as using Fmoc-AA-OH: T3P®: [1.2:6.0 eq.], pH~9 with DIEA, 30 min in 2Me-THF. *In-situ* Fmoc removal (with 16.0 eq. piperidine). Precipitation after Fmoc removal with ACN (1x10 mL) and EtOAc (3x10 mL).

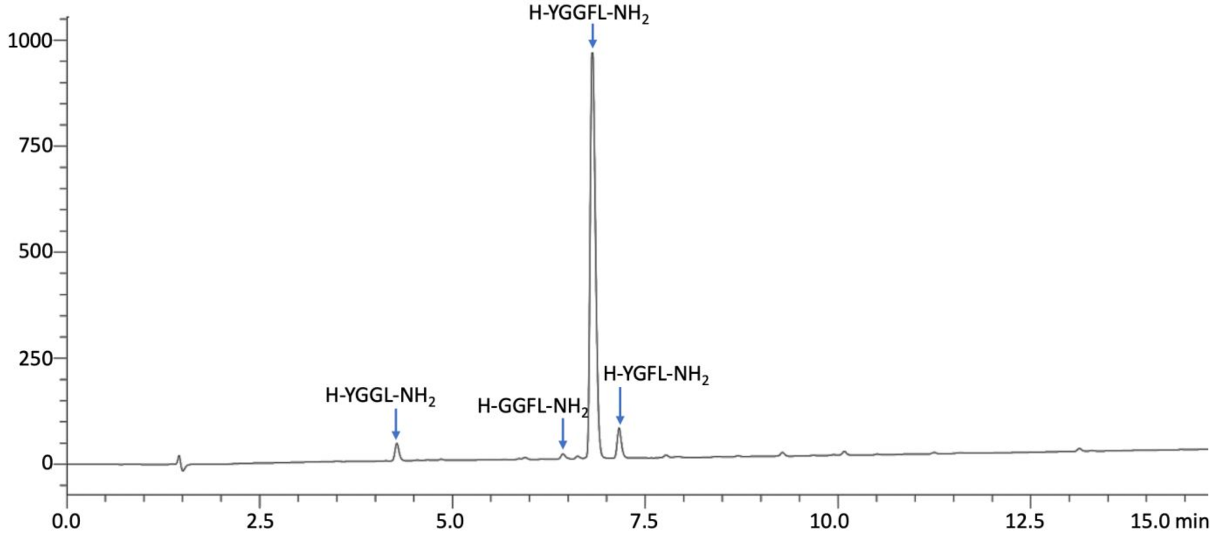

Figure 22. HPLC analysis (using HPLC Method A: 5-60% B (ACN) into A (0.1% TFA in H_2O) in 15 min with flowrate 1 mL/min at 220 nm) of the product obtained by LPPS of Leu-Enkephalin pentapeptide (H-YGGFL-NH_2) as using Fmoc-AA-OH: T3P®: [1.2:8.0 eq.], pH~9 with DIEA, 30 min in 2Me-THF. *In-situ* Fmoc removal (with 16.0 eq. piperidine). Precipitation after Fmoc removal with ACN (1x10 mL) and EtOAc (3x10 mL).

Synthesis of H-Y(*t*Bu)GGFL-RinkAmide-Cyclover using 6.0 eq. of T3P® and DCM:

H-RinkAmide-Cyclover (100 mg, 0.067 mmol, 1.0 eq.) was dissolved in DCM (2 mL). Fmoc-Leu-OH (29 mg, 0.080 mmol, 1.2 eq.), T3P® (50% in EtOAc, 201 µL, 0.4 mmol, 6.0 eq.), and DIEA (140 µL, 0.8 mmol, 12.0 eq.) were added, and the reaction mixture was stirred at room temperature for 30 min. Reaction completion was monitored by TLC (*n*-hexane/EtOAc = 7:3) as explained above. After completion of the coupling, piperidine (106 µL, 1.07 mmol, 16.0 eq.) was added directly to the reaction mixture for *in-situ* Fmoc deprotection. The mixture was stirred at room temperature for 30 min, and deprotection was confirmed by TLC (*n*-hexane/EtOAc = 7:3) as explained above. After completion of the reaction, as confirmed by TLC and HPLC, ACN (10 mL) was added to the reaction mixture to induce precipitation. The mixture was sonicated for 1 min and then centrifuged, and the supernatant was discarded. The resulting precipitate was washed with EtOAc (10 mL), sonicated for 1 min, and centrifuged. Supernatant was decanted. This EtOAc (10 mL) washing step was repeated twice to obtain the H-L-RinkAmide-Cyclover. Subsequently, chain elongation was continued by sequential coupling of the Fmoc-Phe-OH (32 mg, 0.08 mmol, 1.2 eq.) Fmoc-Gly-OH (24 mg, 0.08 mmol, 1.2 eq.) Fmoc-Gly-OH (24 mg, 0.08 mmol, 1.2 eq.) Fmoc-Tyr(*t*Bu)-OH (37 mg, 0.08 mmol, 1.2 eq.), T3P® (201 µL, 0.4 mmol, 6.0 eq.), and DIEA (140 µL, 0.8 mmol, 12.0 eq.) in DCM (2 mL). After each coupling step piperidine (106 µL, 1.07 mmol, 16.0 eq.) was added directly to the reaction mixture for *in-situ* Fmoc deprotection. After each *in-situ* Fmoc removal step Cyclover-tagged peptide was isolated by precipitation with ACN (10 mL) and EtOAc (3x10 mL). The final product was dried under *vacuo* to afford H-Y(*t*Bu)GGFL-RinkAmide-Cyclover as a white solid (127 mg, 93% yield).

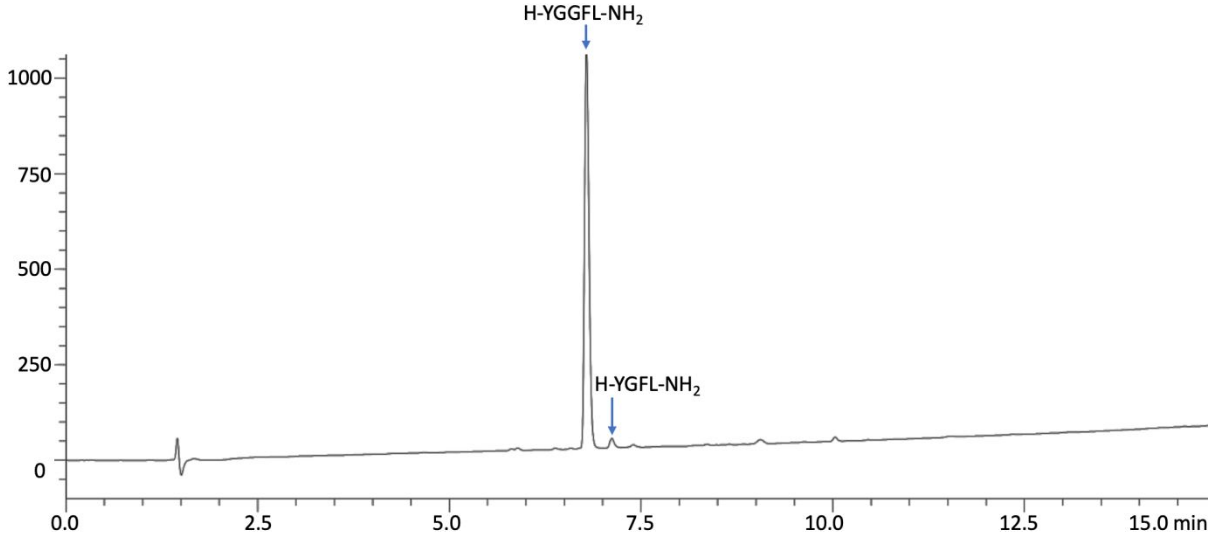

Figure 23. HPLC analysis (using HPLC Method A: 5-60% B (ACN) into A (0.1% TFA in H_2O) in 15 min with flowrate 1 mL/min at 220 nm) of the product obtained by LPPS of Leu-

Enkephalin pentapeptide (H-YGGFL-NH$_2$) as using Fmoc-AA-OH: T3P®: [1.2:6.0 eq.], pH~9 with DIEA, 30 min in DCM. *In-situ* Fmoc removal (with 16 eq. piperidine). Precipitation after Fmoc removal with ACN (1x10 mL) and EtOAc (3x10 mL).

Scheme 2. Synthesis of H-YGGFL-NH$_2$ *via* Extraction Approach

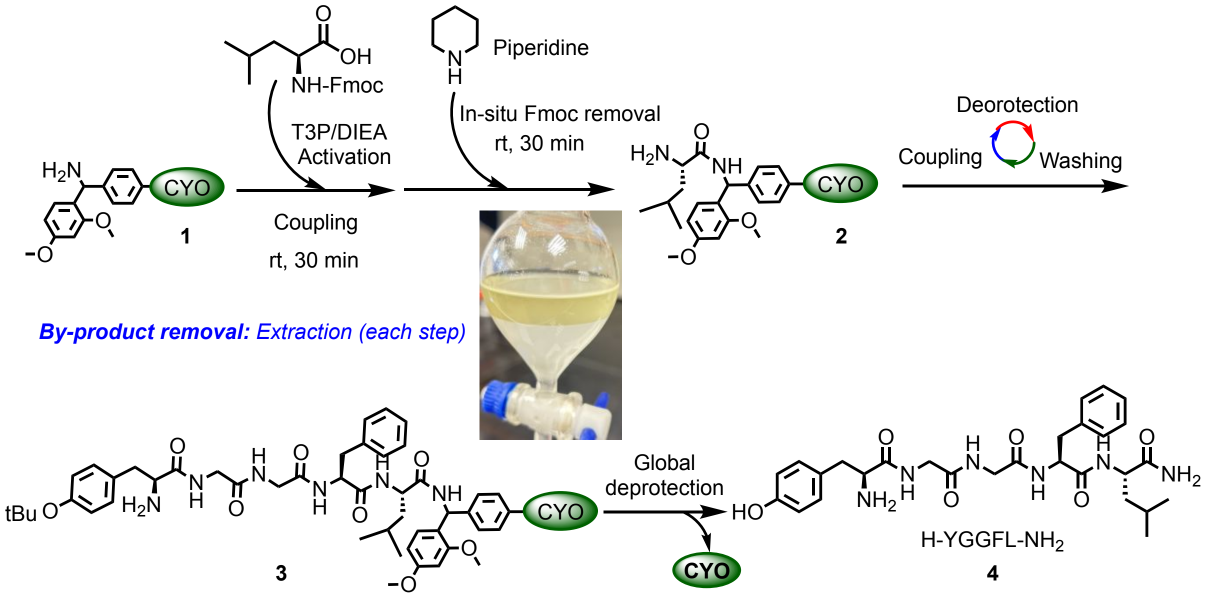

Synthesis using Extraction Approach: H-RinkAmide-Cyclover (100 mg, 0.067 mmol, 1.0 eq.) was dissolved in 2-MeTHF (2 mL). Fmoc-Leu-OH (29 mg, 0.080 mmol, 1.2 eq.), T3P® (50% in EtOAc, 268 µL, 0.54 mmol, 8.0 eq.), and DIEA (187 µL, 1.07 mmol, 16.0 eq.) were added. The mixture was stirred at room temperature for 30 min until completion (*n*-hexane/EtOAc = 7:3) as explained above. After that, piperidine (212 µL, 2.144 mmol, 32.0 eq.) was added directly to remove the Fmoc group. The mixture was stirred for 30 min at room temperature, and deprotection was confirmed by TLC (*n*-hexane/EtOAc = 7:3) as explained above. After completion of the reaction, the reaction mixture was extracted with 0.1 N HCl (2 mL). The organic (2-MeTHF) layer was further extracted with aqueous NaHCO$_3$ (2 mL) and then with brine solution (2 mL). The organic layer was then dried over anhydrous MgSO$_4$ (1 g) and filtered. The compound H-L-RinkAmide-Cyclover, along with the unreactive DBF-piperidine adduct, remained in the organic phase, while aqueous-soluble impurities (H-Leu-OH, T3P® by-product, excess base) were removed during the extraction. The organic layer was directly used for the next reaction. Subsequently, chain elongation was carried out by sequential coupling of Fmoc-Phe-OH (32 mg, 0.08 mmol, 1.2 eq.), Fmoc-Gly-OH (24 mg, 0.08 mmol, 1.2 eq.), Fmoc-Gly-OH (24 mg, 0.08 mmol, 1.2 eq.), and Fmoc-Tyr(*t*Bu)-OH (37 mg, 0.08 mmol, 1.2 eq.) to the Cyclover-tagged peptide using T3P® (268 µL, 0.54 mmol, 8.0 eq.), and

DIEA (187 µL, 1.07 mmol, 16.0 eq.). After each coupling step, piperidine (212 µL, 2.144 mmol, 32.0 eq.) was added directly to the reaction mixture for *in-situ* Fmoc deprotection. Following each deprotection step, the Cyclover-tagged peptide intermediate was isolated by extraction methods as explained above. At the final step, ACN (10 mL×1) and was added to the solution of H-Y(*t*Bu)GGFL-RinkAmide-Cyclover to remove the DBF-piperidine adduct. The mixture was sonicated for 1 min and centrifuged, and the supernatant was discarded. The resulting precipitate was washed with EtOAc (10 mL×2), sonicated, and centrifuged. The final product was dried under *vacuo* to afford H-Y(*t*Bu)GGFL-RinkAmide-Cyclover as a white solid (129 mg, 95% yield).

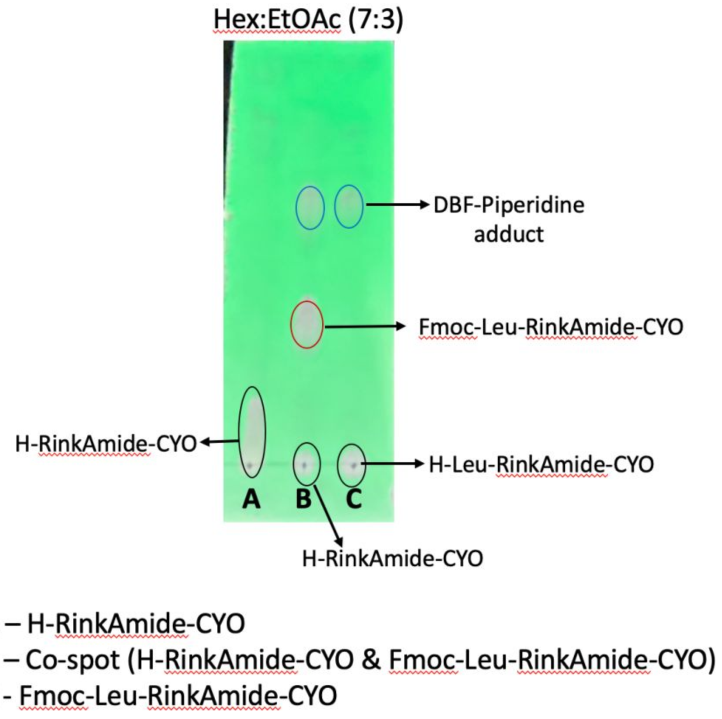

Figure 24. TLC analysis of Fmoc-Leu-RinkAmide-Cyclover *via* extraction method: **A)** H-RinkAmide-CYO; **B)** Co-spot of H-RinkAmide-CYO and Fmoc-L-RinkAmide-CYO using 8.0 eq. T3P®; **C)** H-L-RinkAmide-CYO

Global Deprotection and Isolation of H-YGGFL-NH$_2$ (4) from Cyclover:

Compound H-Y(*t*Bu)GGFL-RinkAmide-Cyclover (129 mg) was dissolved in a cleavage cocktail of TFA:TIS:DCM (95:2.5:2.5, 1 mL) and stirred at room temperature for 2 h. After completion, TFA amount was reduced, and TBME (10 mL, 10-fold excess to reaction mixture) was added to the residue to precipitate the peptide. The mixture was centrifuged, the supernatant discarded, and the precipitate was washed twice with TBME to remove residual

cleavage reagents and Cyclover. The resulting crude peptide was dried under *vacuo* to yield H-YGGFL-NH_2 as a solid (34 mg, 93%).

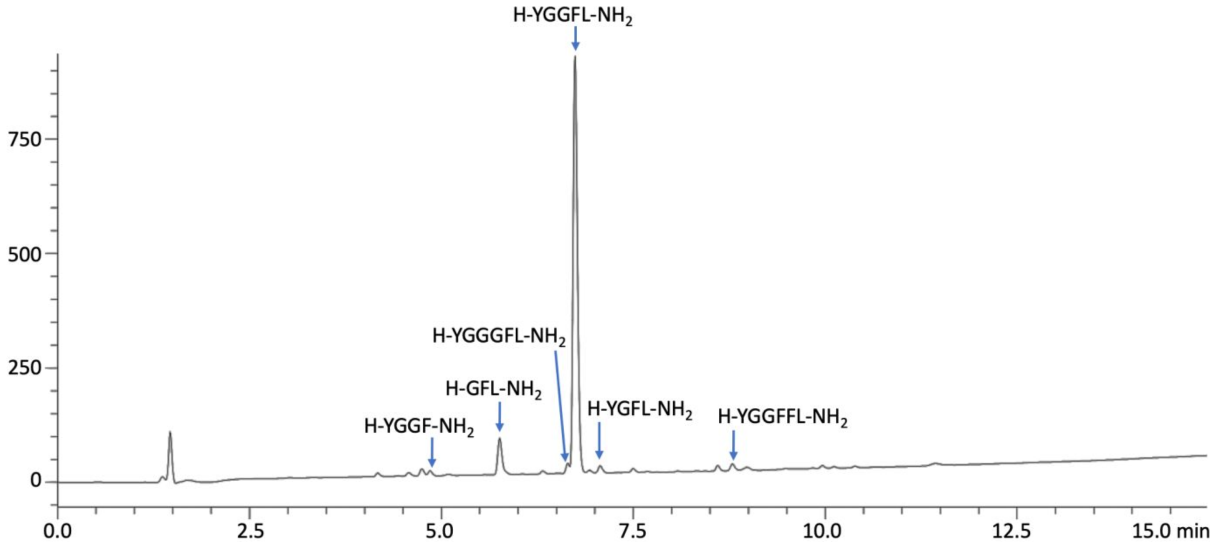

Figure 25. HPLC analysis (using HPLC Method A: 5-60% B (ACN) into A (0.1% TFA in H_2O) in 15 min with flowrate 1 mL/min at 220 nm) of the product obtained by LPPS of Leu-Enkephalin pentapeptide (H-YGGFL-NH_2) as using Fmoc-AA-OH: T3P®: [1.2:6.0 eq.], pH~9 with DIEA, 30 min in 2Me-THF. *In-situ* Fmoc removal (with 32 eq. piperidine). Extraction after Fmoc removal with 0.1N HCl, $NaHCO_3$ and brine.

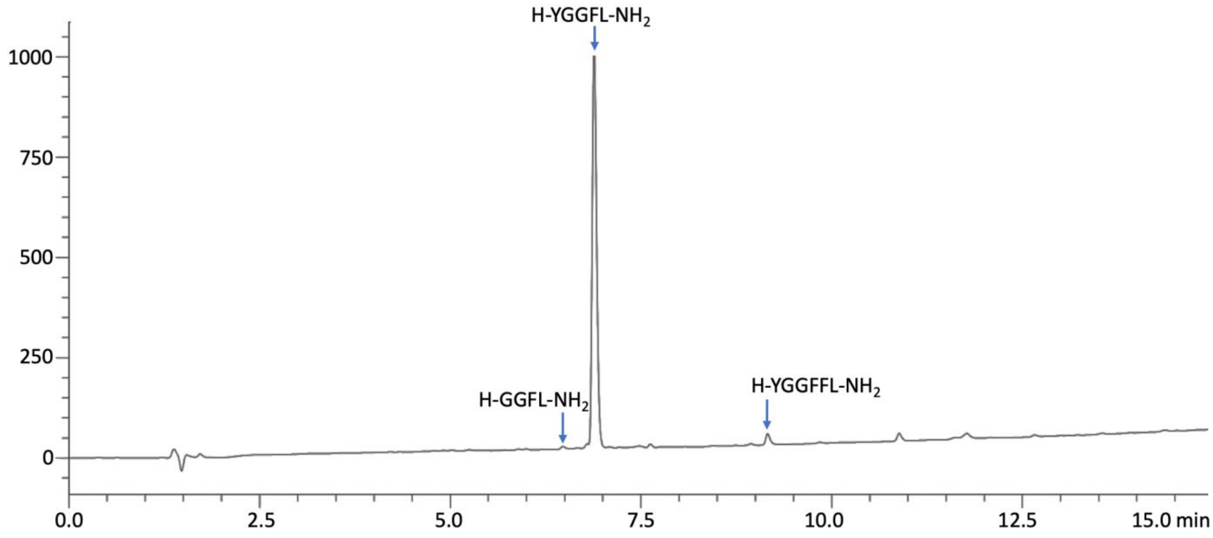

Figure 26. LPPS of Leu-Enkephalin pentapeptide (H-YGGFL-NH_2) as using Fmoc-AA-OH: T3P®: [1.2:8.0 eq.], pH~9 with DIEA, 30 min in 2Me-THF. *In-situ* Fmoc removal (with 32 eq. piperidine). Extraction after Fmoc removal with 0.1N HCl, $NaHCO_3$ and brine. HPLC using Method A (5-60% B (ACN) into A (0.1% TFA in H_2O) in 15 min with flowrate 1 mL/min at 220 nm).

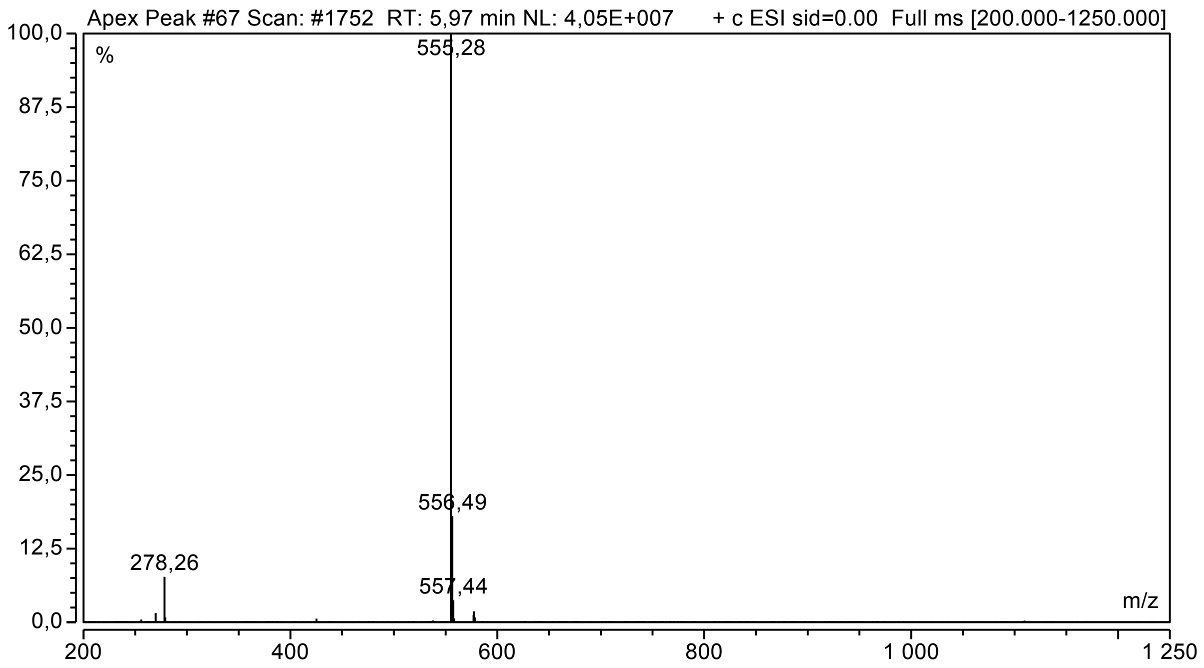

Figure 27. LCMS for Leu-enkephalin. MS (ESI) m/z: $[M+H]^+$ Calcd for $C_{28}H_{39}N_6O_6$ 555.29; Found 555.28.

Scheme 3. Synthesis of Oxytocin H-C(Acm)YIQNC(Acm)PLG-NH_2 (7) *via* Precipitation/Extraction Approach

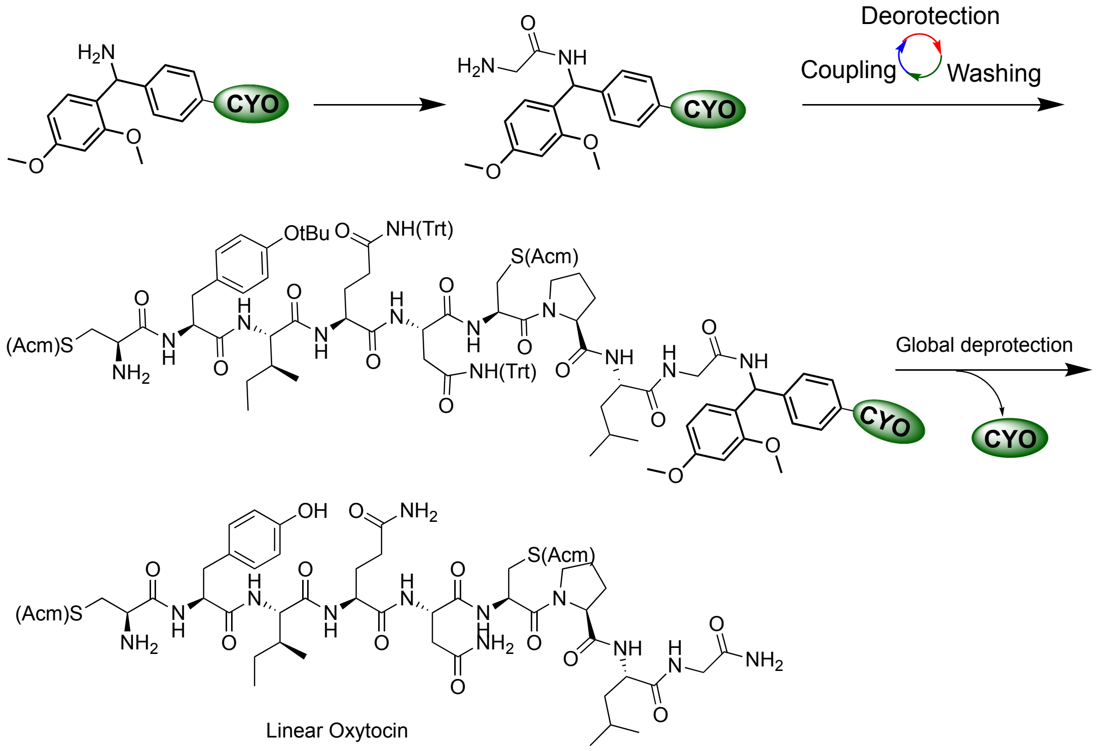

Oxytocin H-C(Acm)YIQNC(Acm)PLG-NH_2 (7) Synthesis Using Precipitation Approach: H-RinkAmide-Cyclover (100 mg, 0.067 mmol, 1.0 eq.) was dissolved in 2-MeTHF (2 mL). Fmoc-Gly-OH (24 mg, 0.080 mmol, 1.2 eq.), T3P® (50% in EtOAc, 268 µL, 0.54 mmol, 8.0 eq.), and DIEA (187 µL, 1.07 mmol, 16 eq.) were added. The mixture was stirred at room temperature for 30 min until completion (*n*-hexane/EtOAc = 7:3) as explained above. After completion of the coupling, piperidine (106 µL, 1.07 mmol, 16 eq.) was added directly to the reaction mixture for *in-situ* Fmoc deprotection. The mixture was stirred at room temperature for 30 min, and deprotection was confirmed by TLC (*n*-hexane/EtOAc = 7:3) as explained above. After completion of the reaction, as confirmed by TLC and HPLC, ACN (10 mL) was added to the reaction mixture to induce precipitation. The mixture was sonicated for 1 min and then centrifuged, and the supernatant was discarded. The resulting precipitate was washed with EtOAc (10 mL), sonicated for 1 min, and centrifuged. Supernatant was decanted. This EtOAc (10 mL) washing step was repeated twice to obtain the H-Gly-RinkAmide-Cyclover. Subsequently, chain elongation was carried out by sequential coupling of Fmoc-Leu-OH (29 mg, 0.08 mmol, 1.2 eq.), Fmoc-Pro-OH (27 mg, 0.08 mmol, 1.2 eq.), Fmoc-Cys(Acm)-OH (33 mg, 0.08 mmol, 1.2 eq.), Fmoc-Asn(Trt)-OH (48 mg, 0.08 mmol, 1.2 eq.), Fmoc-Gln(Trt)-OH (49 mg, 0.08 mmol, 1.2 eq.), Fmoc-Ile-OH (29 mg, 0.08 mmol, 1.2 eq.), Fmoc-Tyr(tBu)-OH

(37 mg, 0.08 mmol, 1.2 eq.) and Fmoc-Cys(Acm)-OH (33 mg, 0.08 mmol, 1.2 eq.) to the Cyclover-tagged peptide using T3P® (50% in EtOAc, 268 μL, 0.54 mmol, 8.0 eq.), and DIEA (187 μL, 1.07 mmol, 16 eq.). After each coupling step piperidine (106 μL, 1.07 mmol, 16.0 eq.) was added directly to the reaction mixture for *in-situ* Fmoc deprotection. After each *in-situ* Fmoc removal step Cyclover-tagged peptide was isolated by precipitation with ACN (10 mL×1) and EtOAc (10 mL×3). The final product was dried under *vacuum* to afford H-C(Acm)Y(*t*Bu)IQ(Trt)N(Trt)C(Acm)PLG-RinkAmide-Cyclover as a white solid (166 mg, 86% yield).

Oxytocin H-C(Acm)YIQNC(Acm)PLG-NH_2 (7) Synthesis Using Extraction Approach: H-RinkAmide-Cyclover (100 mg, 0.067 mmol, 1.0 eq.) was dissolved in 2-MeTHF (2 mL). Fmoc-Gly-OH (24 mg, 0.080 mmol, 1.2 eq.), T3P® (50% in EtOAc, 268 μL, 0.54 mmol, 8.0 eq.), and DIEA (187 μL, 1.07 mmol, 16 eq.) were added. The mixture was stirred at room temperature for 30 min until completion (*n*-hexane/EtOAc = 7:3). After that, piperidine (212 μL, 2.144 mmol, 32.0 eq.) was added directly to remove the Fmoc group. The mixture was stirred for 30 min at room temperature, and deprotection was confirmed by TLC (*n*-hexane/EtOAc = 7:3) as explained above. After completion of the reaction, the reaction mixture was extracted with 0.1 N HCl (2 mL). The organic layer (2-MeTHF) was further extracted with aqueous $NaHCO_3$ (2 mL) and then with brine solution (2 mL). The organic layer was then dried over anhydrous $MgSO_4$ (1 g) and filtered. The compound H-Gly-RinkAmide-Cyclover, along with the unreactive DBF-piperidine adduct, remained in the organic phase, while aqueous-soluble impurities (H-Gly-OH, T3P® by-product, excess base) were removed during the extraction. The organic layer was directly used for the next reaction. Subsequently, chain elongation was carried out by sequential coupling of Fmoc-Leu-OH (29 mg, 0.08 mmol, 1.2 eq.), Fmoc-Pro-OH (27 mg, 0.08 mmol, 1.2 eq.), Fmoc-Cys(Acm)-OH (33 mg, 0.08 mmol, 1.2 eq.), Fmoc-Asn(Trt)-OH (48 mg, 0.08 mmol, 1.2 eq.), Fmoc-Gln(Trt)-OH (49 mg, 0.08 mmol, 1.2 eq.), Fmoc-Ile-OH (29 mg, 0.08 mmol, 1.2 eq.), Fmoc-Tyr(tBu)-OH (37 mg, 0.08 mmol, 1.2 eq.) and Fmoc-Cys(Acm)-OH (33 mg, 0.08 mmol, 1.2 eq.) to the Cyclover-tagged peptide using T3P® (268 μL, 0.54 mmol, 8.0 eq.), and DIEA (187 μL, 1.07 mmol, 16.0 eq.). After each coupling step, piperidine (212 μL, 2.144 mmol, 32.0 eq.) was added directly to the reaction mixture for *in-situ* Fmoc deprotection. Following each deprotection step, the Cyclover-tagged peptide intermediate was isolated by extraction methods as explained above. At the final step, ACN (10 mL) was added to the solution of H-Y(*t*Bu)GGFL-RinkAmide-Cyclover to remove the DBF-piperidine adduct. The mixture was sonicated for 1 min and centrifuged, and the

supernatant was discarded. The resulting precipitate was washed with EtOAc (10 mL), sonicated for 1 min, and centrifuged. The supernatant was decanted, and the EtOAc washing step was repeated twice. The final product was dried under *vacuo* to afford H-C(Acm)Y(*t*Bu)IQ(Trt)N(Trt)C(Acm)PLG-RinkAmide-Cyclover as a white solid (172 mg, 90% yield).

Global Deprotection and Extraction of H-C(Acm)Y(tBu)IQ(Trt)N(Trt)C(Acm)PLG-NH$_2$ (7) from Cyclover: H-C(Acm)Y(*t*Bu)IQ(Trt)N(Trt)C(Acm)PLG-RinkAmide-Cyclover (100 mg) was dissolved in a cleavage cocktail consisting of TFA:TIS:H_2O (95.0:2.5:2.5) and stirred at room temperature for 2 h. After completion of the reaction, TFA amount was reduced. Cold TBME (10 mL, 10-fold excess) was added to the residue to precipitate the peptide. The mixture was centrifuged, and the supernatant was discarded. The resulting precipitate was washed twice with TBME (10 mL) and centrifuged again to remove residual cleavage reagents and Cyclover. The resulting crude peptide was dried under *vacuo* to yield crude H-C(Acm)YIQNC(Acm)PLG -NH$_2$ as a white solid in 86% yield *via* precipitation approach and 87% *via* extraction approach.

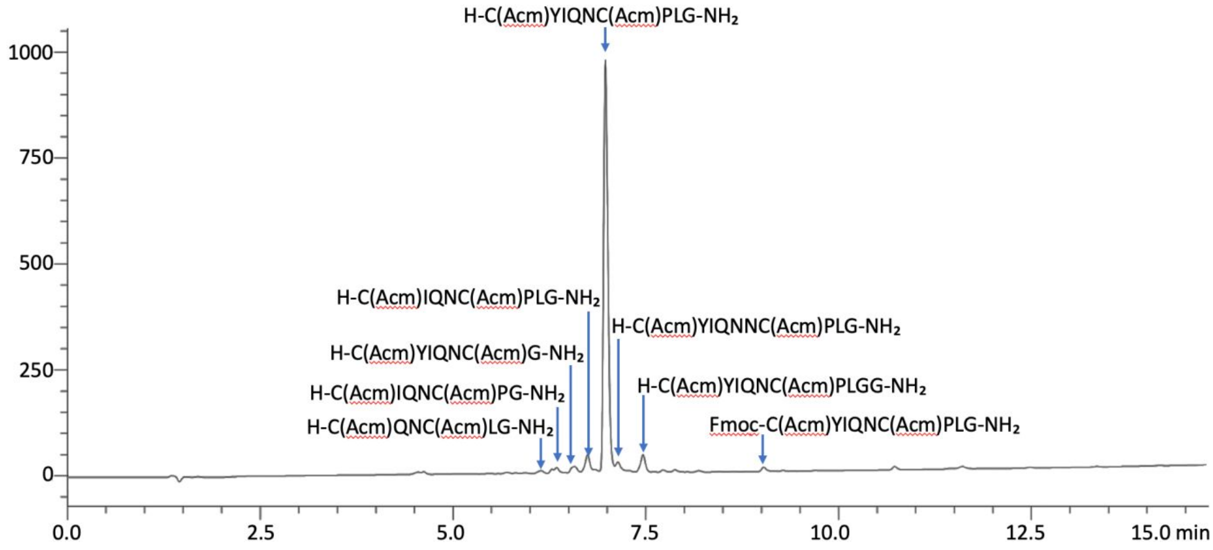

Figure 28. HPLC analysis (using HPLC Method B: 0-60% B (ACN) into A (0.1% TFA in H_2O) in 15 min with flowrate 1 mL/min at 220 nm) of linear oxytocin (C(Acm)YIQNC(Acm)PLG -NH$_2$) as using Fmoc-AA-OH: T3P®: [1.2:8.0 eq.], pH~9 with DIEA, 30 min in 2-MeTHF. *In-situ* Fmoc removal (with 16.0 eq. piperidine). Precipitation after Fmoc removal with ACN (1x10 mL) and EtOAc (3x10 mL).

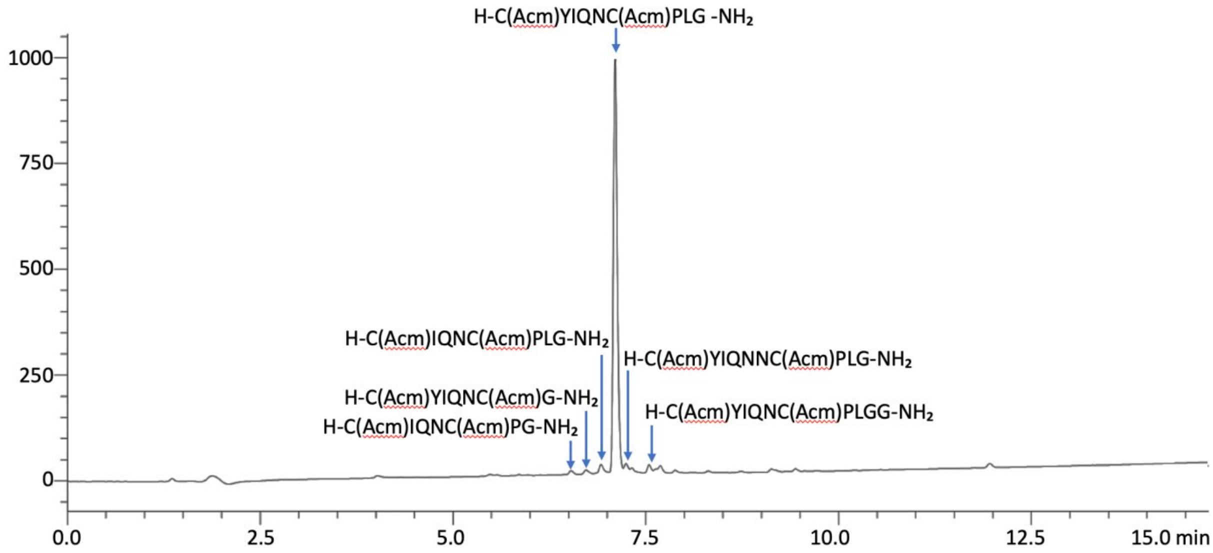

Figure 29. HPLC analysis (using HPLC Method B: 0-60% B (ACN) into A (0.1% TFA in H_2O) in 15 min with flowrate 1 mL/min at 220 nm) of linear oxytocin (C(Acm)YIQNC(Acm)PLG $-NH_2$) as using Fmoc-AA-OH: T3P®: [1.2:8.0 eq.], pH~9 with DIEA, 30 min in 2Me-THF. *In-situ* Fmoc removal (with 32 eq. piperidine). Extraction after Fmoc removal with 0.1N HCl, $NaHCO_3$ and Brine.

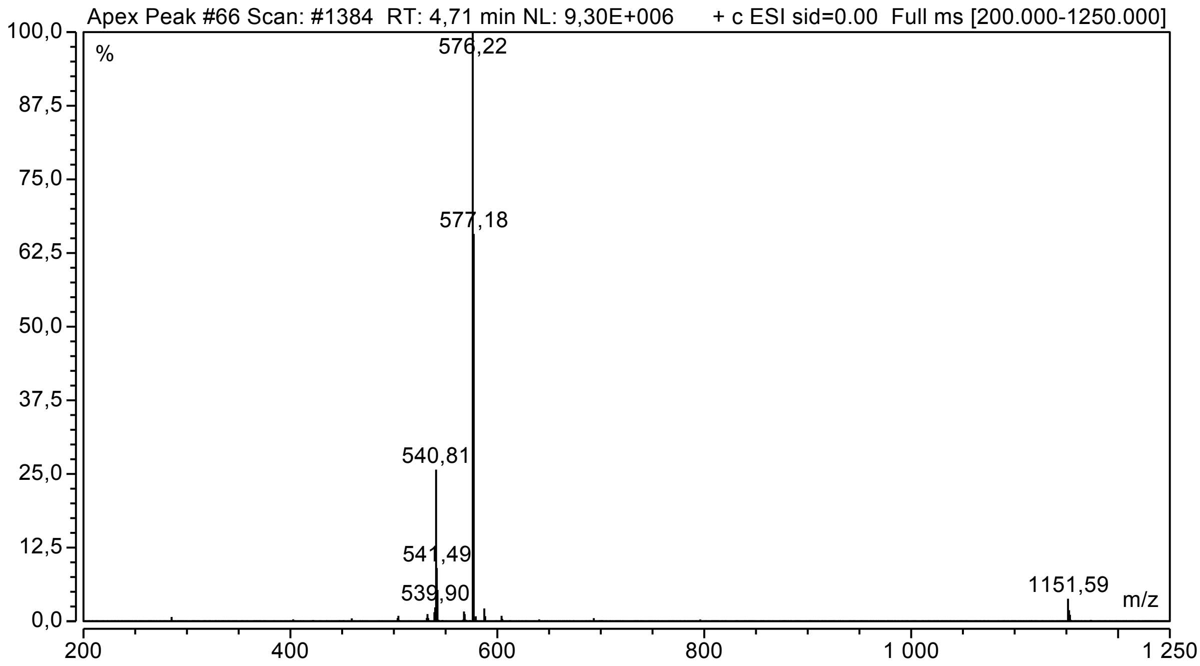

Figure 30. LCMS data of linear oxytocin. MS (ESI) m/z: $[M+H]^+$ Calcd for $C_{49}H_{79}N_{14}O_{14}S_2$ 1151.53; Found 1151.59.

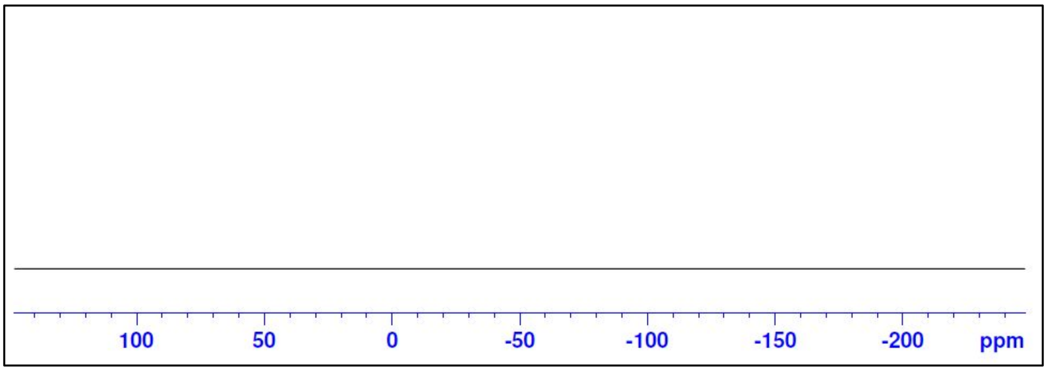

Figure 31. ^{31}P NMR of the Rink attached cyclover which does not contain any trace of T3P® or related byproducts.

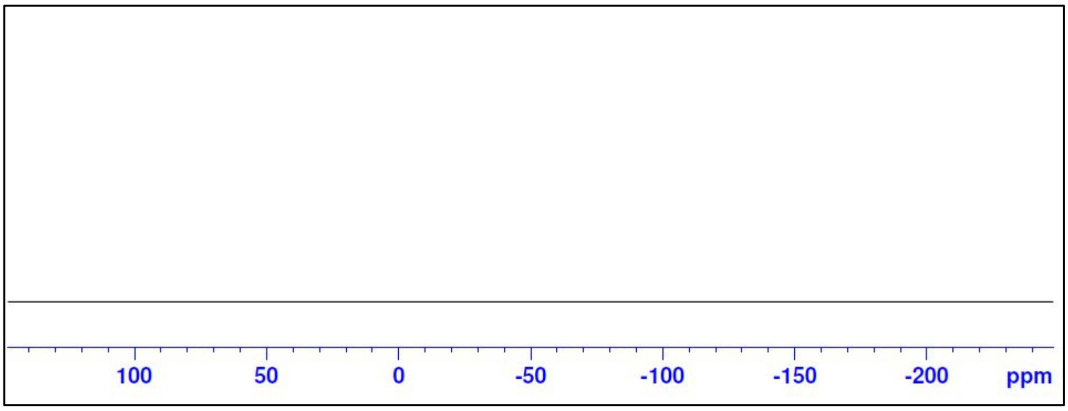

Figure 32. ^{31}P NMR of the Leu-enkephalin peptide which does not contain any trace of T3P® or related byproducts.

References:

(1) Kumar, A.; Sharma, A.; de la Torre, B. G.; Albericio, F. *In Situ* Fmoc Removal: A Sustainable Solid-Phase Peptide Synthesis Approach. *Green Chem.* **2022**, *24* (12), 4887–4896.

(2) Mthethwa, N.; Nandhini, K. P.; Kumar, A.; Sharma, A.; de la Torre, B. G.; Albericio, F. Toward a Sustainable Solid-Phase Peptide Synthesis Strategy: *In Situ* Fmoc Removal. *Green Chem. Lett. Rev.* **2024**, *17* (1), 2325993.

(3) Mattellone, A.; Corbisiero, D.; Ferrazzano, L.; Cantelmi, P.; Martelli, G.; Palladino, C.; Tolomelli, A.; Cabri, W. Speeding up Sustainable Solution-Phase Peptide Synthesis Using T3P® as a Green Coupling Reagent: Methods and Challenges. *Green Chem.* **2023**, *25* (7), 2563–2571.
